# Low-dose brain radiation: lowering hyperphosphorylated-tau without increasing DNA damage or oncogenic activation

**DOI:** 10.1038/s41598-023-48146-w

**Published:** 2023-11-30

**Authors:** Diego Iacono, Erin K. Murphy, Cheryl D. Stimpson, Daniel P. Perl, Regina M. Day

**Affiliations:** 1 DoD/USU Brain Tissue Repository and Neuropathology Program, Uniformed Services University (USU), Bethesda, MD USA; 2 Department of Neurology, F. Edward Hébert School of Medicine, Uniformed Services University (USU), Bethesda, MD USA; 3 Department of Pathology, F. Edward Hébert School of Medicine, Uniformed Services University (USU), Bethesda, MD USA; 4 Neuroscience Program, Department of Anatomy, Physiology and Genetics, F. Edward Hébert School of Medicine, Uniformed Services University (USU), Bethesda, MD USA; 5 The Henry M. Jackson Foundation for the Advancement of Military Medicine (HJF) Inc., Bethesda, MD USA; 6 Neurodegeneration Disorders Clinic, National Institute of Neurological Disorders and Stroke, NINDS, NIH, Bethesda, MD USA; 7 Department of Pharmacology and Molecular Therapeutics, F. Edward Hébert School of Medicine, Uniformed Services University (USU), Bethesda, MD USA

**Keywords:** Neuroscience, Neurology

## Abstract

Brain radiation has been medically used to alter the metabolism of cancerous cells and induce their elimination. Rarely, though, brain radiation has been used to interfere with the pathomechanisms of non-cancerous brain disorders, especially neurodegenerative disorders. Data from low-dose radiation (LDR) on swine brains demonstrated reduced levels of phosphorylated-tau (CP13) and amyloid precursor protein (APP) in radiated (RAD) versus sham (SH) animals. Phosphorylated-tau and APP are involved in Alzheimer’s disease (AD) pathogenesis. We determined if the expression levels of hyperphosphorylated-tau, 3R-tau, 4R-tau, synaptic, intraneuronal damage, and DNA damage/oncogenic activation markers were altered in RAD versus SH swine brains. Quantitative analyses demonstrated reduced levels of AT8 and 3R-tau in hippocampus (H) and striatum (Str), increased levels of synaptophysin and PSD-95 in frontal cortex (FCtx), and reduced levels of NF-L in cerebellum (CRB) of RAD versus SH swine. DNA damage and oncogene activation markers levels did not differ between RAD and SH animals, except for histone-H3 (increased in FCtx and CRB, decreased in Str), and p53 (reduced in FCtx, Str, H and CRB). These findings confirm the region-based effects of sLDR on proteins normally expressed in larger mammalian brains and support the potential applicability of LDR to beneficially interfere against neurodegenerative mechanisms.

## Introduction

It is well known that in biological organisms, and especially mammals, the damage produced by single or fractionated high-dose radiation (HDR) exposure on normal tissues can generate severe acute and chronic consequences^1–3^. Based on the total dose of radiation adsorbed and specific organ-tissue radiosensitivity characteristics, post-irradiation consequences on body organs, and brain in particular, can be life-threatening or culminate in relevant and therapeutically challenging clinical situations^4^.

By contrast, much less has been investigated in terms of possible short- and long-term effects of low-dose radiation (LDR) exposure, especially γ-radiation exposure, on normal brain tissues and non-cancerous brain illnesses such as neurodegenerative disorders.

While a few studies have been performed^5^, more systematic investigations are needed to identify either detrimental or beneficial effects caused by sLDR (i.e., < 2.0 Gy total dose) in larger normal mammalian brains using molecular techniques such as antibody-based Western blotting (WB) protein expression quantification, polymers-based immunohistochemistry (IHC), double- and triple-immunofluorescence (IF) protocols, qPCR, -omics, and others^6^. In fact, most of the prior investigations studying the effects of LDR on normal brain tissues only used small mammals (mainly rodents)^7–9^, limited surgically-resected human brain samples^10^ or in vitro simplified experimental models (i.e., neuronal and glial cells cultures)^11^.

To better judge the applicability of LDR experimental findings obtained through commonly used small lab animals (i.e., rodents) to humans, larger animals, such as swine, are actually intermediate-sized mammals well suited to acquire, confirm or expand our knowledge on the various effects of LDR on normal organs and tissues, including brain and brain-related tissues (spinal cord, eyes, inner ears, peripheral nerves, etc.). Indeed, it has been extensively demonstrated that swine represent an extremely valuable tool of research to study a wide variety of human conditions^12^. In terms of metabolic, cardio-circulatory, cognitive, behavioral, immune and pharmacological aspects, swine are indeed much more similar to humans when compared to rodents^13^.

Recently, we reported some surprising findings in large animals (swine) demonstrating that after 28 days from a total body single low-dose radiation exposure (sLDR; 1.79 Gy, 0.485–0.502 Gy/min), the expression levels of phosphorylated-tau (pTau/CP13) and amyloid precursor protein (APP) were reduced in specific regions of the normal brain: namely, Frontal cortex (FCtx), Hippocampus (H), and Cerebellum (CRB)^14^. The relevance of these new findings are connected to the fact that both pTau and APP are molecules known to be directly involved in the pathogenesis of neurodegenerative disorders, and Alzheimer’s disease (AD) in particular^15^. Based on these unforeseen effects of sLDR in the brain, and in some specific regions of it, sLDR appears to be a biologically plausible tool for the selective, and perhaps region-based, reduction of proteins pathologically-accumulated in large mammalian brains, including human subjects with a diagnosis of AD, and other misfolding protein-linked diseases.

Here, we aimed to further investigate the effects of sLDR across different regions of normal swine brains, by measuring the expression levels of some other more AD- and non-AD-specific (although still neurodegeneration associated) proteins in radiated (RAD) versus sham (SH) swine 28 days after a sLDR whole body exposure (1.79 Gy, 0.485–0.502 Gy/min).

Specifically, we aimed to:

- Determine if the previously described sLDR-induced protein expression lowering effect on CP13 (pTau) could also occur for other Tau proteins more characteristically linked to AD pathomechanisms such as hyperphosphorylated-tau (pTau; AT8), three-repeat tau (3R-tau) and four-repeat tau (4R-tau). As reminder, 3R-tau/4R-tau ratios change during the natural progression of AD and other neurodegenerative disorders^16^;
- Measure if non-AD-specific brain proteins, that is proteins not only involved in AD but also in other neurodegenerative processes, such as pre-, post-, spino-, synaptic (synaptophysin, PDS95, spinophilin), and intraneuronal damage (neurofilament light chain [NF-L]) markers could be similarly changed in RAD versus SH swine;
- Measure if the expression levels of cellular stress response, DNA damage, and oncogenic activation markers such as p53, capsase-3, cleaved-caspase-3, pCHK2 and histone-H3 differed in RAD versus SH swine in general as well as in those same brain regions in which some of the above-described markers (point a and b) resulted altered.

Moreover, in comparison to our previous study, the current investigation assessed additional brain regions that included: Frontal cortex (FCtx), Hippocampus (H), Striatum (Str), Thalamus-Hypothalamus (Thal/Hypothal) and Cerebellum (CRB).

## Results

### Neurodegeneration-associated protein expression levels

**Figure 1:**
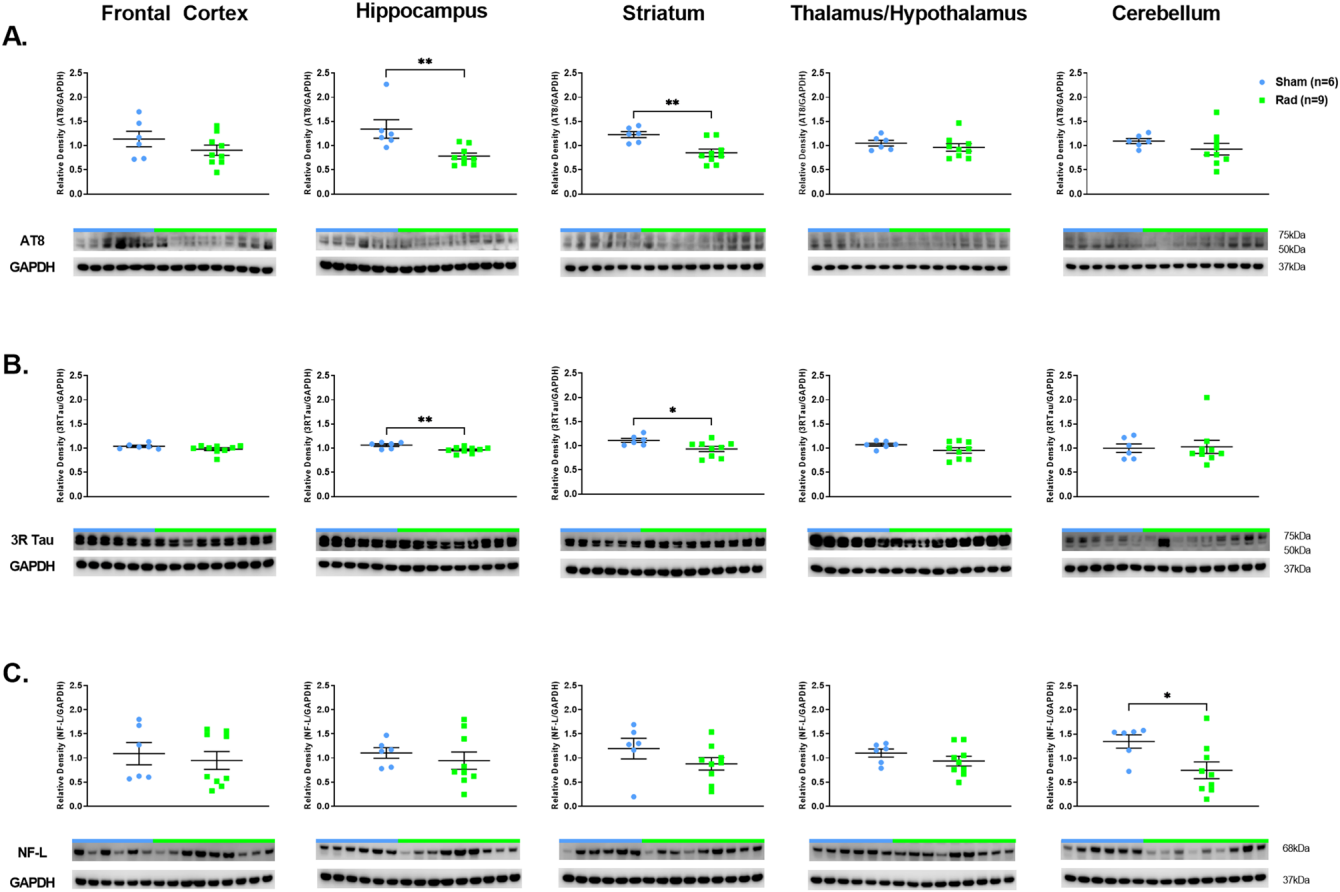
Western blot densitometric analysis and representative blots for AT8 (**A**), 3RTau (**B**) and NF-L (**C**) in Frontal Cortex, Hippocampus, Striatum, Thalamus/Hypothalamus and Cerebellum from the brains of Sham (blue) and Irradiated (green) swine 33–35 days post total body radiation. * indicates *p* < 0.05 and ** indicates *p* < 0.01 as determined by unpaired 2-tailed *t*-test. Error bars represent the standard error of the mean. Each blot was run in duplicate and the graphs represent the average of 2 runs. Original full-length blots are presented in Supplementary Figs. 3–5.

The analyses showed lower expression levels of pTau (AT8) in the H (*p* = 0.0062, df = 13) and Str (*p* = 0.0042, df = 13) of RAD versus SH swine (Fig. 1A). Likewise, lower levels of 3R-Tau in the H (*p* = 0.009, df = 13) and Str (*p* = 0.032, df = 13) of RAD versus SH swine were detected (Fig. 1B). 4R-Tau expression levels showed a trend toward lower expression levels across both H and Str, but the results were not significant between RAD versus SH animals (Supplemental Fig. 1A). Lower expression level of NF-L in the CRB of RAD versus SH swine (*p* = 0.03, df = 13) was detected (Fig. 1C).

### Synaptic-associated protein expression levels

**Figure 2:**
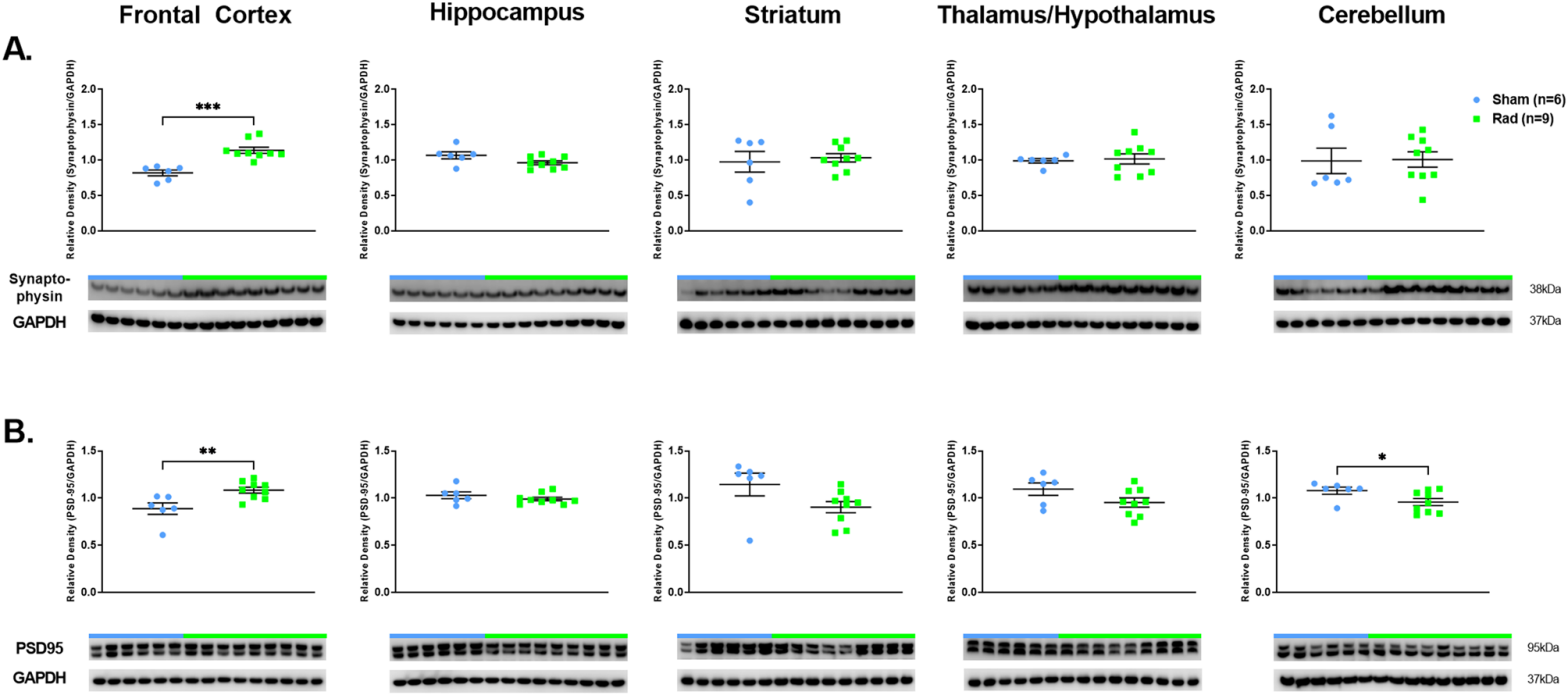
Western blot densitometric analysis and representative blots for Synaptophysin (**A**), PSD95 (**B**) in Frontal Cortex, Hippocampus, Striatum, Thalamus/Hypothalamus and Cerebellum from the brains of Sham (blue) and Irradiated (green) swine 33–35 days post total body radiation. * indicates *p* < 0.05, ** indicates *p* < 0.01 and *** indicates *p* < 0.001 as determined by unpaired 2-tailed *t*-test. Error bars represent the standard error of the mean. Each blot was run in duplicate and the graphs represent the average of 2 runs. Original full-length blots are presented in Supplementary Figs. 6–7.

WB analyses showed higher levels of SYN (*p* = 0.0002, df = 13) and PSD95 (*p* = 0.0076, df = 13) in the FCtx, and lower levels of PSD95 (*p* = 0.049, df = 13) in the CRB of RAD versus SH (Fig. 2A and B). No changes in SPINO expression levels across all examined regions were observed (Supplemental Fig. 1B).

### DNA-damage-associated protein expression levels

**Figure 3:**
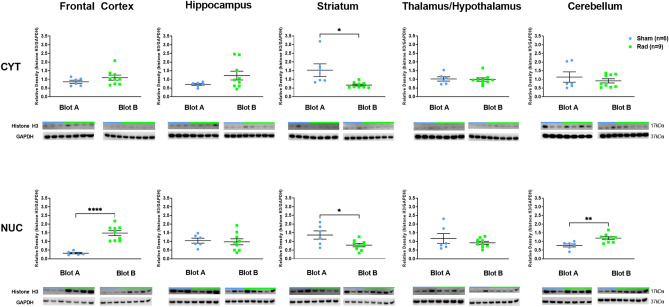
Western blot densitometric analysis and representative blots for Histone-H3 in Cytosolic (top) and Nuclear (bottom) extracts from Frontal Cortex, Hippocampus, Striatum, Thalamus/Hypothalamus and Cerebellum from the brains of Sham (blue) and Irradiated (green) swine 33–35 days post total body radiation. *indicates *p* < 0.05, **indicates *p* < 0.01 and *** indicates *p* < 0.001 as determined by unpaired 2-tailed *t*-test. Error bars represent the standard error of the mean. It required 2 blots (A and B) to run the complete set of Cytosolic and Nuclear samples at the same time and each of these were run in duplicate. The graphs represent the average of 2 runs. Original full-length blots are presented in Supplementary Figs. 8–12.

**Figure 4:**
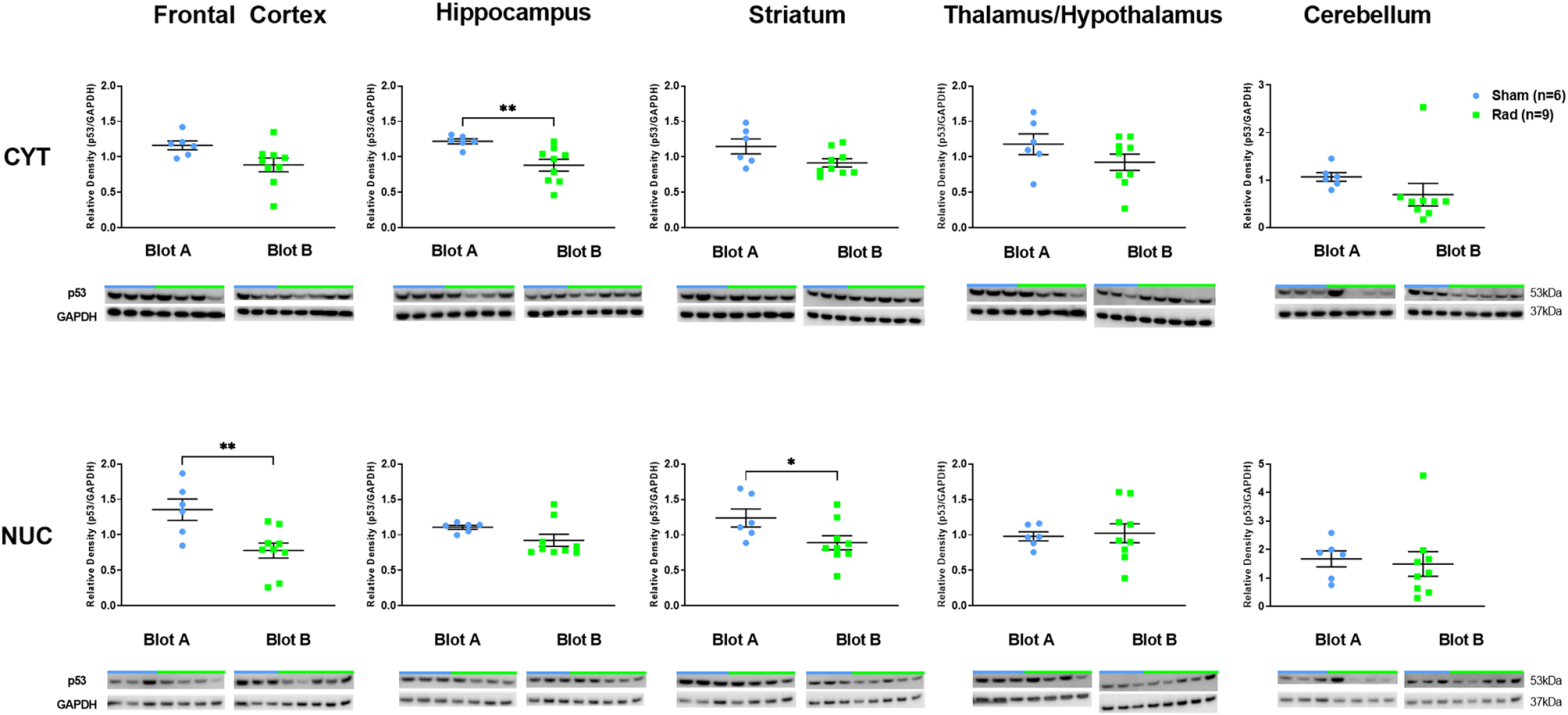
Western blot densitometric analysis and representative blots for p53 in Cytosolic (top) and Nuclear (bottom) extracts from Frontal Cortex, Hippocampus, Striatum, Thalamus/Hypothalamus and Cerebellum from the brains of Sham (blue) and Irradiated (green) swine 33–35 days post total body radiation. *indicates *p* < 0.05 and **indicates *p* < 0.01 as determined by unpaired 2-tailed *t*-test. Error bars represent the standard error of the mean. It required 2 blots (**A** and **B**) to run the complete set of Cytosolic and Nuclear samples at the same time and each of these were run in duplicate. The graphs represent the average of 2 runs. Original full-length blots are presented in Supplementary Figs. 13–17.

There were no expression level changes observed in WB for caspase-3, cleaved-caspase-3, or pCHK2 in RAD versus SH across all examined regions (Supplemental Fig. 2A–C). However, p53 and Histone-H3 expression levels showed differences at cytosolic and nuclear levels in specific regions of RAD versus SH swine brains. Decreased expression levels of histone-H3 at both cytosolic (*p* = 0.0132, df = 13) and nuclear (*p* = 0.0215, df = 13) levels in Str and at nuclear level in FCtx (*p* < 0.0001, df = 13) and CRB (*p* = 0.0046, df = 13) in RAD versus SH swine were measured (Fig. 3). Decreased levels of cytosolic-p53 in the H (*p* = 0.008, df = 13), as well as of nuclear-p53 in the FCtx (*p* = 0.0068, df = 13) and Str (*p* = 0.047, df = 13) of RAD versus SH animals were detected (Fig. 4).

### IHC and IF findings

**Figure 5:**
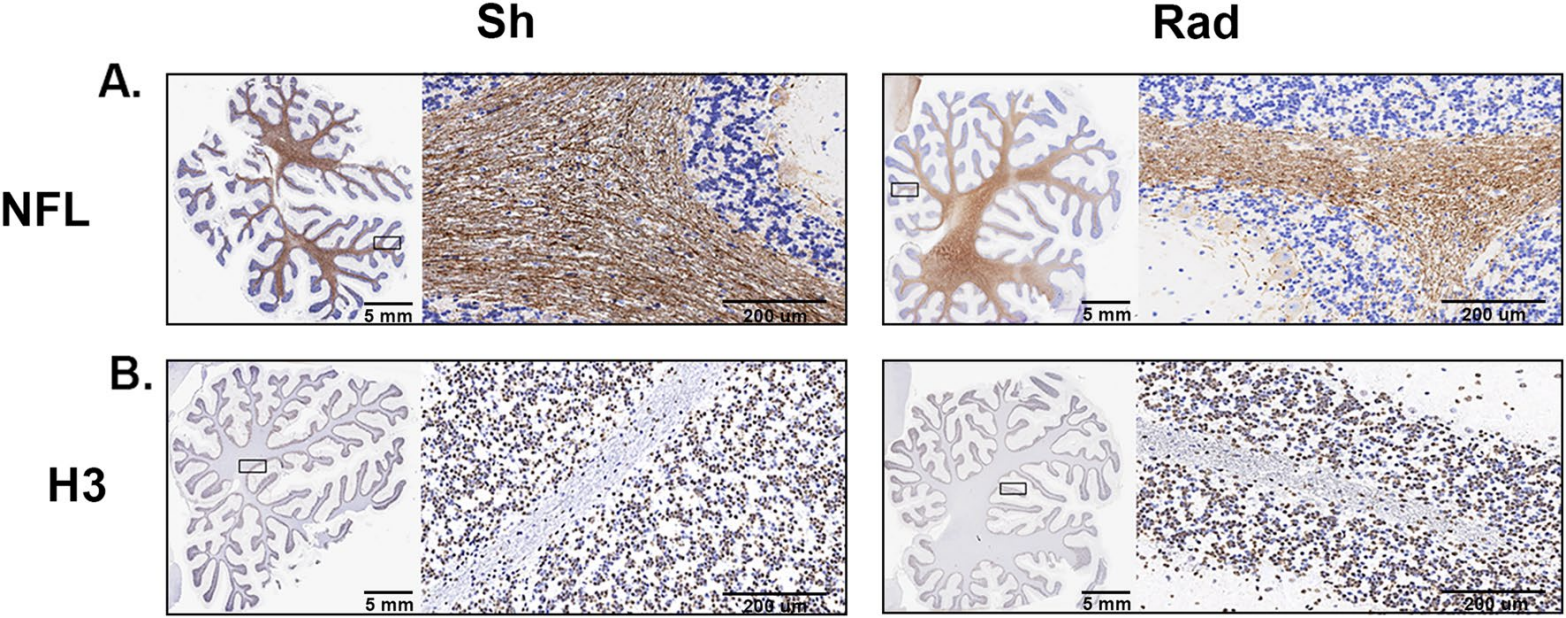
The figure shows swine Cerebellum sections stained by immunohistochemistry (IHC) methods for neurofilament light chain (NFL) (**A**) and histone-H3 (**B**) in radiated (RAD) versus sham (SH) animals.

IHC qualitative assessment confirmed reduced IHC-reactivity for NF-L in the white matter of CRB of RAD versus SH (Fig. 5A). However, as also indicated by WB outcomes, no IHC NF-L changes, based on a qualitative assessment, were detectable in the FCtx of the corresponding swine brains where cerebellar NF-L qualitative changes were found. Also, the qualitative assessment of IHC for histone-H3 stain in the CRB was not able to detect differences (Fig. 5B).

**Figure 6:**
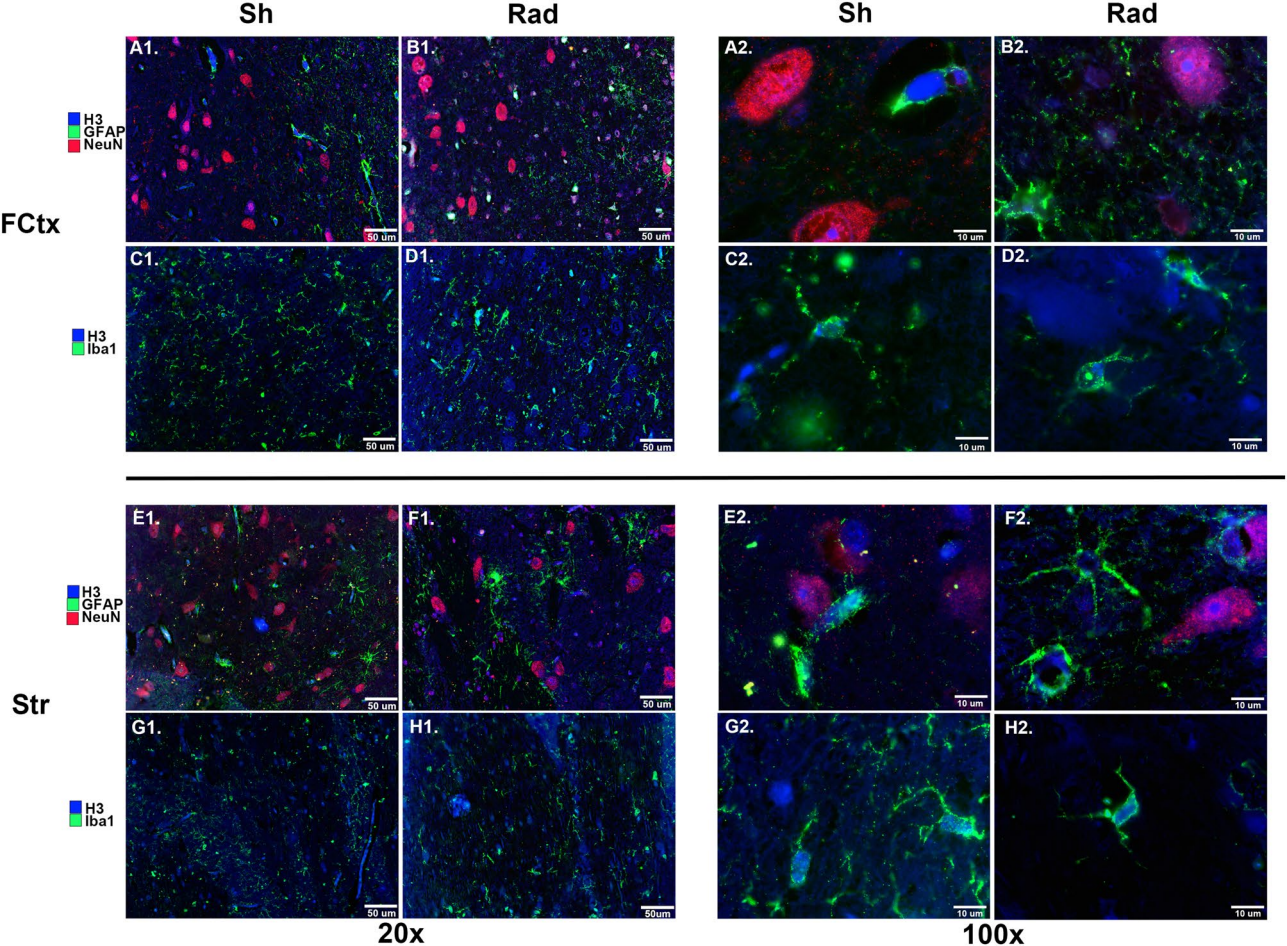
The figure shows Frontal Cortex (FCtx, panels **A**_**1–2**_**-D**_**1–2**_) and Striatum (Str, panels **E**_**1–2**_**-H**_**1–2**_) of control versus irradiated swine. **A**_**1**_**–H**_**1**_ demonstrate the widespread immunostaining at 20X while **A**_**2**_**-H**_**2**_ images allow for better visibility of individual cells at 100X. **A**_**1–2**_**, B**_**1–2**_**, E**_**1–2**_**, F**_**1–2**_ are triple labeled with H3 (blue), GFAP (green), and NeuN (red). **C**_**1–2**_**, D**_**1–2**_**, G**_**1–2**_**, H**_**1–2**_ are double labeled with H3 (blue) and Iba1 (green). Scale is 50 µm (20x) and 10 µm (100x).

IF qualitative assessment of histone-H3 in the FCtx and Str—the specific brain regions where significant changes in expression level were detected by WB analyses—confirmed that histone-H3 is a molecule normally expressed in neurons, astroglial, and microglial cells of the swine cerebral cortex. While quantitative measures of histone-H3 were outside the scope of the current investigation, the triple IF-stain, histone-H3 + NeuN + GFAP, confirmed the WB-based outcomes that showed a higher IF-reactivity of histone-H3 in the nucleus of FCtx neurons of RAD versus SH swine with unchanged levels of IF-reactivity of histone-H3 in astroglial cells of the same region (Fig. 6, A1–B1 and A2–B2**)**. Also, the double IF-stain, histone-H3 + IBA1, suggested that cortical microglial cells were not primarily involved in histone-H3 expression changes as neurons appear to be (Fig. 6, C1–D1 and C2–D2). Consistent with WB results, lower levels of IF-reactivity were found for cytoplasmic and nuclear histone-H3 in striatal neurons versus astroglial and microglial cells of RAD versus SH brains (Fig. 6, E1–H1 and E2–H2).

## Discussion

In civilian and military contexts, there are numerous reasons why the correct and precise identification of short- and long-term effects produced by single (sLDR) or repeated LDR (rLDR) exposure on body organs, and brain in particular, using larger normal mammals is currently critical^17^. For example, in clinical neuro-oncology, it is well recognized that cognitive deficits may occur after a mid-term period (months) from cranial/brain radiation^18–21^. These cognitive effects are especially important when related to the long-term impact on pediatric populations, which are more often the types of populations affected by brain radiotherapy-related consequences. Another fundamental reason to more accurately investigate sLDR or r-LDR effects on body organs dwells in the currently diffuse use of LDR apparatuses for security checking purposes, medical screenings for the general population and the potential use of LDR as a tactical nuclear weapon in war or terroristic scenarios.

Additionally, the now existing technological capacities for space travel provides another important reason to more precisely investigate the possible long-term consequences of LDR on normal biological tissues, especially on the central nervous and cardiovascular systems. In fact, galactic cosmic radiation (GCR)—which also includes a discrete percentage of γ-rays—is more than a potential risk for future long-term astronauts and space travelers. Altogether, these diverse modalities of radiation exposure demand an increased rate of investigation especially those aiming to analyze all possible detrimental or beneficial short- and long-term effects of LDR on mammalian organs^22,23^.

Our prior results demonstrated that a sLDR exposure of 1.79 Gy after 28 days is able to reduce, in specific regions of normal swine brains, a set of proteins normally expressed in the cerebral tissue, which are also involved in pathomechanisms activated during sporadic neurodegenerative processes, such as AD pathomechanisms. Deepening those surprising sLDR outcomes on the normal brain tissues, these additional data have confirmed and extended those initial findings and further support the potential beneficial application of sLDR on disease-specific brain regions in order to induce expression level reduction of those proteins typically associated with neuronal loss and cognitive dysfunction processes (i.e. accumulation of AT8-neurofibrillary tangles in hippocampal pyramidal neurons and memory disorders in AD).

Here, as additional novelty, we illustrate that some of the more specific AD-related proteins such as hyperphosphorylated-Tau (AT8) and 3R-tau, are significantly decreased in the H and Str of RAD versus SH after 28 days from a sLDR. Furthermore, most of the synaptic expression levels were unchanged across most of the regions assessed, and if changes were detected, they were directed toward an increased expression level in RAD versus SH animals, representing then, potentially, another unexpected and beneficial effect, at least in swine, of sLDR in specific areas of the brain such as the Frontal cortex (Results, Fig. 2A and B).

Our data demonstrating an sLDR-induced effect on the reduction of AT8 expression in brain regions such as the H and Str are actually of particular relevance considering the fact that these two neuroanatomical regions, and especially the H, are among those brain regions largely involved in the accumulation of AT8-NFTs in AD. In support of this notion, recent animal studies showed that low levels of ionizing energy were indeed able to reduce AD-like pathology, mainly *β*-amyloid lesions, in transgenic mice^24^. These rodent studies have triggered the possibility to validate the use of LDR in human subjects, especially AD subjects, characterized by higher levels of *β*-amyloid accumulation. However, while studies in rodents are extremely valuable, it would be necessary and safer to validate those results performing similar and even more detailed experiments in larger animals such as swine and non-human primates.

Also, more in general, the mechanisms of Tau reduction still remain to be precisely identified. In speculative terms, we hypothesize two main possible mechanisms: one mechanism consisting in a “direct pathway” that is, a direct effect of ionizing energy on the microtubule associated protein tau (MAPT) gene—either at genomic/epigenomic or regulatory factors level—and another mechanism consisting in an “indirect pathway” that is, the reduction of Tau levels would be driven mainly by isolated or co-occurring mechanisms of autophagy, ubiquitin–proteasome or other metabolic processes that still need to be identified.

Intriguingly, the expression levels for most of the DNA damage and oncogenic activation markers such as caspase-3, cleaved-caspase-3 and pCHK2 were not different in RAD versus SH swine across all brain regions examined. As a reminder, caspase-3 and cleaved-caspase-3 are among the best-known apoptotic-inducing molecules identified and often activated in many oncogenic, injury-related processes, and neuronal death processes too^25^. However, during the last decade, it has been observed that caspase-3, and its derivative and interconnected molecules, have some physiological function especially in terms of synaptic neuroplasticity during either the developmental or adult life period^26–29^. In this context, it seems that sLDR does not determine significant changes of caspase-3 activities and its related physiological function. At least in a short-/mid-term post-radiation period. However, future age-related/dose–effect studies will be necessary to confirm this initial observation.

Moreover, histone-H3 and p53 expression levels showed differences in RAD versus SH in specific regions of the normal swine brain. Histone-H3 is one of the most “*sensitive*” proteins for chromosomic and DNA translational modifications, which allows the coordinated and timely expression of specific genes in tissues, including brain tissue, as related to specific functions or metabolic needs of each tissue, organ, or functional region of it. However, histone-H3 has been primarily studied in the context of genetic mutations and oncogenesis processes and much less is acknowledged about its function, or dysfunction, during normal brain developmental, adulthood, aging, and neuroplasticity processes^30^. More in general though, we speculate that histone-H3 modifications are tightly linked to the typical molecular features of each brain region, for example Striatum versus Frontal Cortex, to specifically respond or compensate for the effects of LDR in relationship to their circuital function or metabolism. In fact, differences between cellular compartments (i.e. nucleus vs. cytoplasm marker levels) and cytoplasmic-nucleus molecular trafficking could be proxy signals for more complex molecular processes. However, most of these complex molecular processes need to be still precisely identified across normal development, normal aging and pathological conditions in mammals and humans in particular.

Based on our IF qualitative analyses, histone-H3 is present in the nuclei of neurons, astroglial, and microglial cells (Fig. 6). Our IF light microscopy-based assessment demonstrated that a major impact of sLDR on histone-H3 expression levels in neuronal cells in comparison to astroglial and microglia cells in the same region (see FCtx and Str in Fig. 6). This new finding implies that histone-H3 reacts relatively early after sLDR and its neuronal activation could be part of LDR-related neuroadaptive processes. The possibility that histone-H3 is part of neuroplasticity phenomena has been proposed in studies across several animal species^31,32^.

We also detected alterations in p53 expression levels in RAD versus SH swine. p53 is mostly known for its role as a tumor suppressor protein, but recent findings suggest that it plays pleiotropic roles in neuroadaptive and neuroreparative mechanisms^33,34^. In addition, p53 seems to modulate either beneficial or detrimental functioning based on specific types of stress and specific types of neural cells involved. It is now recognized that modulation of p53 during brain development is associated with programmed neuronal death as part of normal brain developmental mechanisms^35^. In addition, recent findings have shown that reduced p53 expression levels are associated with an increased neuronal survival when associated with calcium-binding synaptic modulation^36^ or with the amelioration of neuromuscular junction loss without affecting motor neuron pathology^37^. In contrast, p53 activation in microglial cells seems to be a detrimental factor for the synapses during inflammation^38^. For example, p53 loss in oligodendrocytes provided protection against subcortical white matter damage and recognition memory impairment in a white matter stroke model^39^. Moreover, various animal studies demonstrated that p53 is elevated during the initial phases of AD and other neurodegenerative conditions^40–44^. Potentially then, sLDR on the entire brain, or a specific region of it, could have also some possible advantageous effect in terms of p53 reduction in the context of the natural progression of AD and likely other neurodegenerative processes.

Furthermore, we showed that NF-L levels are reduced in the CRB of RAD versus SH swine. NF-L has been proposed as a plasma biomarker for different neurological conditions (AD, ALS, multiple sclerosis, spino-cerebellar ataxias, etc.). We could not confirm the presence of higher levels of NF-L in the plasma of RAD versus SH animals (a higher plasma level of NF-L is assumed to be correlated with brain NF-L loss as signal of brain injury). However, recent investigations expressed some concerns on the reliability of serum/plasma NF-L measures as direct correlate of cognitive performance, fatigue, depression or anxiety in MS patients^45^ or ALS patients across all stages of the disease^46^. These recent data underline the complexity and non-linearity between intracerebral (and possibly intraregional) and peripheral levels of NF-L across different neurological disorders and brain circuits. It is also possible that reduced expression levels of NF-L represent a transitory phenomenon related to neuroadaptive events linked to the specific role of NF-L in the brain^47^, and more specifically the CRB, or specific cerebellar cellular types. In fact, due to specific metabolic difference in comparison to the other regions of the brain, the CRB could be particularly radio-sensitive to γ-radiation^48^ and may be a brain region to avoid for possible radiotherapeutic interventions in AD patients.

Intriguingly, previous studies in rodents and non-human primates showed not only that LDR does not induce AD-like pathogenesis^49^ but cognition can actually improve^50,51^. Moreover, other types of radiation such as ion-radiation showed sex-specific (female) reduction of amyloid and gliosis in mice^52^, and possibly positive counterbalancing effects of galactic cosmic rays (GCR) have been hypothesized in astronauts^53^.

Systematic low-dose/low dose rate radiation-brain effect investigations using larger mammals such as swine and AD-swine models in particular are needed. This current situation is partially due to historical reasons for which there has been a constant concern about the use of radiation for neurotherapeutic use, even at low dose or low dose rates, based on the commonly accepted assumption that there is a linear non-threshold (LNT) effect between radiation adsorbed and biological detrimental effect. Remarkably, though, newer and consistent amounts of data do not entirely support this view about the global validity of the LNT model and corollary assumptions^54,55^. Actually, new experimental analyses and large epidemiological studies that analyzed big datasets, evidenced that γ-radiation effects do not necessarily follow the simple LNT paradigm under certain radiation doses or dose/rates conditions, especially below certain low dose or dose rate thresholds^56–59^.

Our new data seem to confirm the possible intrinsic hormetic nature of γ-radiation, especially in terms of its interactions with normal adult mammalian brain tissue. Moreover, these interactions seem to be related to the intrinsic radio-sensitivity of certain neuroanatomical regions (for example, Frontal cortex vs. Striatum vs. Cerebellum). If these novel results will be confirmed by the controlled use of sLDR across different disease-specific animal models, in either small or larger animals^56^, the above described modulatory effects of sLDR on brain molecules in specific brain regions or neurocircuits, could be applied in specific clinical contexts such as AD treatment. Furthermore, this cranial, or more likely focal, neuro-radiotherapeutic approach may have a broad applicability for other neurodegenerative, or psychiatric conditions as well, after diligent exclusion of possible major short- and long-term detrimental effects, if any.

As a limitation of this study, we would like to point out that γ-H2AX analyses were not performed. γ-H2AX is a validated DNA damage marker useful to measure DNA damage, especially acute DNA damage after irradiation. Although most of the γ-H2AX studies have been performed in rodents and much less in larger animals, γ-H2AX remains a useful DNA damage marker used to analyze long-term radio-toxic effects also in combination with other factors such as naturally accumulated DNA damage during aging, neurodegeneration, DNA repair mechanisms-related efficiency across body tissues and cell types^60,61^. Moreover, γ-H2AX seems to react differentially based on multiple factors such as age at time of irradiation (embryonic vs. developmental vs. adult vs. older age) or acute versus chronic irradiation, and it might also have pleiotropic effects across different body systems, tissues, and cell types as well^62^. In our study, the animals were young adult swine exposed to LDR after developmental age and were assessed after more than 4 weeks from irradiation, which are factors to consider when compared to other mammals (i.e. rodents) and conditions (naïve vs. transgenic animals, younger vs. older, etc.)^63^. Interestingly, though, direct correlations between γ-H2AX and cleaved-Caspase 3 have been described^64^. These latter findings, if confirmed, could imply that in our study since there were no significant differences in cleaved Caspase-3 across all examined regions then γ-H2AX levels across the examined brain regions should also remain unchanged. However, more extensive and quantitative analyses measuring possible different levels of γ-H2AX and other DNA damage markers across different brain regions are actually needed to assure that no increase of DNA damage could be induced by LDR to the brain tissue or specific cells of the CNS.

## Methods

All methods and procedures have been described in Iacono et al.^14^. Refer to that article for methods details and experimental design. Below, we provide only a brief description for the animal use and methods relevant and specifically employed for the current investigation.

### Animal use

All animal handling procedures followed the guidelines from the National Research Council for the ethical handling of laboratory animals and approved by the Uniformed Services University (USU) and Armed Forces Radiobiology Research Institute (AFRRI) Institutional Animal Care and Use Committees (Protocol PHA-18-942). Additionally, all animal procedures followed the PHS Policy on Humane Care and Use of Laboratory Animals, the NIH Guide for the Care and Use of Laboratory Animals, all applicable Federal regulations governing the protection of animals in research as well as in compliance with the ARRIVE guidelines (https://arriveguidelines.org/arrive-guidelines). Male Göttingen minipigs ranging in age from ~ 5.0–6.5 months and weighing 8–11 kg at the time of arrival, were purchased from Marshall Farms Group Ltd. USA (North Rose, NY, USA). Animals were kept in a barrier facility accredited by the Association for Assessment and Accreditation of Laboratory Animal Care International. Animals were pair housed. Housing rooms were maintained at 21 ± 2 °C, 50 ± 10% humidity, and 12-h light/dark cycle with food and water available ad libitum. Minipigs were divided into the following two groups:

1. Sham (SH) (n = 6)
2. Radiation (RAD) (n = 9)

Approximately two weeks after arrival, minipigs receiving total-body radiation were deeply anesthetized with Telazol/Xylazine (4.4–2 mg/kg) and transported to the High-Level Cobalt facility at AFRRI. While anesthetized, animals were positioned in supportive slings and exposed one at a time, bilaterally, to a target total body dose of 1.79 Gy of Cobalt (60Co) radiation delivered at a dose rate of 0.485–0.502 Gy/min, as previously described^14^. After radiation procedures, each animal was transported back to the housing facility for recovery. Minipigs assigned to the sham (SH) groups were also deeply anesthetized with the same dose of Telazol/Xylazine in the housing facility but were not transported to the Cobalt facility.

### Tissue collection

Thirty-three (33) to thirty-five (35) days post radiation or sham procedure, euthanasia was performed with an intracardial injection of Euthasol (4.5 ml/kg) and confirmed by lack of heartbeat. Each animal underwent necropsy procedures by a trained veterinary pathologist for the sampling of organs including the brain and spinal cord. Brains were dissected at the median line of the corpus callosum (CC) to separate the two cerebral and cerebellar hemispheres. The left hemisphere was flash-frozen in chilled liquid isopentane on dry ice. Frozen brains were transferred to − 80 °C until use for protein analyses. The right hemispheres were placed in 10% buffered formalin for tissue fixation to be used for histology.

### Protein extraction and western blot (WB) procedures

The left cerebral hemispheres were cut into 100 µm thick sections in a cryostat and further microdissected into the following 5 anatomical regions: Frontal cortex (FCtx), Hippocampus (H), Striatum (Str), Thalamus and Hypothalamus (Thal/Hypothal), and Cerebellum (CRB). The dissections were guided by following the Göttingen Minipig Brain Atlas (https://www.cense.dk/miniswine_atlas).

Samples containing both gray matter (GM) and subjacent white matter (WM) were homogenized in glass dounce homogenizers with ice cold lysis buffer (1 ml/100 mg tissue; 50 mM Tris–HCl (pH 8), 1% Igepal, 150 mM NaCl, 1 mM EDTA, 1 mM PMSF, 1 mM NaF, 1:100 protease inhibitor cocktail (Sigma-Aldrich, P2714, St. Louis, MO, USA). Samples were centrifuged at 12,000 × g for 20 min to separate the cytosolic (supernatant) and nuclear (pellet) fractions. Supernatants were collected, aliquoted and frozen at − 80 °C. The pellets remaining from the centrifugation were used for nuclear protein extraction. Pellets were resuspended in Nuclear Extraction Buffer (Millipore, 90,498, Billerica, MA, USA) containing 0.5 mM DTT and 1:1000 Protease Inhibitor Cocktail (Sigma-Aldrich, P2714, St. Louis, MO USA) utilizing a motorized pellet mixer. The nuclear suspension was mixed on a rotator at 4 °C for 1 h. Suspension was centrifuged at 16,000 × g for 5 min at 4 °C and the supernatant containing nuclear extract was collected, aliquoted and frozen at − 80 °C. Total protein content from cytosolic and nuclear extracts for each brain region was determined using the Micro BCA assay (Thermo-Fisher Scientific, 23235, Waltham, MA, USA). Cytosolic protein extracts were used for all proteins of interest and nuclear extracts were used for the evaluation of histone-H3 and p53 only. WB were carried out as previously described^14^. All WB were run in duplicate and averaged for each antibody with all protein signal intensities normalized to GAPDH signal intensity. Densitometry was performed with NIH ImageJ software (2.0.0).

### Primary antibodies

The following primary antibodies were used:

- *Neurodegeneration-associated markers* AT8, phosphorylated tau S202 and T205, (1:500; Thermo-Fisher Scientific, MN1020); 3R-tau, 3-repeat isoform of full length tau, (1:1000; Millipore-Sigma, 05–803, Billerica, MA, USA); 4R-tau, 4-repeat isoform of full length tau, (1:1000; Millipore-Sigma, MABN1185, Billerica, MA, USA); neurofilament light chain (NF-L), a marker of neuronal damage, (1:1000, Encor Biotechnology, MCA-6H112, Gainesville, FL, USA).
- *Synaptic markers* Synaptophysin (SYN), a pre-synaptic marker, (1:10,000, abcam, ab8049, Cambridge, MA, USA); PSD95, a post-synaptic marker, (1:5000, Antibodies Inc. 75–028, Davis, CA, USA), Spinophilin (SPINO), a dendritic spine marker, (1:1000, Cell Signaling, 14136, Danvers, MA, USA).
- *DNA-damage markers* p53 (1:1000, MyBioSource, MBS8242548, San Diego, CA, USA); caspase3/cleaved-caspase3 (1:1000, Proteintech, 19677-1-AP, Rosemont, IL, USA); histone-H3 (1:1000, Cell Signaling, 9715, Danvers, MA, USA); pCHK2 (1:1000, Cell Signaling, 2197, Danvers, MA, USA).

### Secondary antibodies

The following HRP tagged secondary antibodies were used: Goat anti-mouse (1:2000, Abcam, ab97040, Cambridge, MA, USA or 1:5000, Proteintech, SA00001-1, Rosemont, IL, USA), Goat anti-rabbit (1:2000, Abcam, ab97080, Cambridge, MA, USA).

### Immunohistochemistry (IHC) and immunofluorescence (IF) procedures

In this investigation, we focused on IHC and IF analyses for NF-L and histone-H3 only in those brain regions where these proteins were found to be differently expressed in RAD versus SH animals based on WB results. Specifically, we performed IHC for NF-L and histone-H3 in the CRB, FCtx and Str, and IF for histone-H3 only in the FCtx and Str.

### Tissue processing, IHC stain, and digitalization procedures

Tissue blocks from each randomly selected animal (*n* = 3 RAD, *n* = 3 SH) were uniformly processed using an automated tissue processor (ASP 6025, Leica Biosystems, Nussloch, Germany). After tissue processing, each tissue block was embedded in paraffin and cut in a series of 20, 5 µm-thick consecutive sections, 1 section per slide. IHC procedures for NFL and Histone-H3 in CRB, FCtx and Str were performed using a Leica Bond III automated immunostainer with a diaminobenzidine chromogen detection system (DS9800, Leica Biosystems, Buffalo Grove, IL). The following antibodies were used: anti-NF-L (1:2000, epitope retrieval time 10 min, Encor Biotechnology, MCA-DA2, Gainesville, FL, USA); anti-Histone-H3 (1:150, epitope retrieval time 10 min Cell Signaling, 9715, Danvers, MA, USA).

All IHC-stained sections were scanned by an Aperio scanner system (Aperio AT2—High Volume, Digital whole slide scanning scanner, Leica Biosystems, Inc., Richmond, IL) and stored in Biolucida system, a hub for 2D and 3D image data (version 2017, MBF Bioscience, Williston, VT, USA) for further assessment and analyses to verify the immunoreactivity (IR) for each antibody and histological distribution of possible lesions and their severity across all examined brain regions and conditions. A microscopic assessment for each region and animal was performed using Aperio ImageScope (Aperio ImageScope, version 2016, Leica Biosystems, Inc.).

### Tissue processing and IF procedures

Based on WB-based histone-H3 expression levels detected across the different brain regions examined and due its typical localization at the nuclear level, we performed a series of IF staining procedures to verify the localization or possible mis-localization of histone-H3 visualized by double and triple IF stains. In particular, we performed a triple IF stain with histone-H3 and neuronal and astroglial markers (histone-H3 + NeuN + GFAP), and double IF stains with histone-H3 and a microglial cell marker (histone-H3 + IBA1) in the FCtx and Str, where expression changes in RAD versus SH animals for this molecule were found. Slides, from the same series used for IHC above, containing sections of FCtx or Str were randomly selected from RAD (n = 4) and SH (n = 3) groups. Sections were deparaffinized in an oven at 60 °C for 45 min, washed 3 times with xylene, and then placed in decreasing percentages of ethanol. Sections were then placed in an EDTA solution (0.37 g EDTA in 1L dH20 with 500uL Tween; pH 9.5) for antigen retrieval and microwaved for 5 min. Tissue was allowed to cool in antigen retrieval buffer for 45 min. Following incubation, sections were rinsed in phosphate-buffered saline (PBS) 3 × 10 min. Sections were pre-blocked in dilution buffer, containing 10% normal serum with 0.4% Triton-X in PBS for 1 h. Tissue was then placed in primary antibody in PBS with 3% normal serum and incubated overnight at 4 °C. Sections from each individual were either triple labeled with anti-rat histone H3 (histone-H3; 1:100, Active Motif, 61,647), anti-mouse glial fibrillary acidic protein (GFAP; 1:250, Leica, PA0026), and anti-rabbit neuron specific nuclear protein (NeuN; 1:500, ProteinTech, 26975-1-AP); or double labeled with anti-rat histone-H3 and anti-rabbit ionized calcium binding adapter (IBA1; 1:500, Genetex, GTX635363). Following overnight incubation, sections were rinsed in PBS and incubated for one h in AlexaFluor (Invitrogen, Carlsbad, CA) secondary antibodies made in goat for fluorophores 405 anti-rat (A48261), 488 anti-mouse (A11029), 488 anti-rabbit (A11034), and 594 anti-rabbit (A11037). Secondary antibodies were used at 1:200 dilutions in PBS containing 3% normal serum. Sections were then rinsed and incubated for 30 min in 0.1% Sudan Black B to reduce autofluorescence. Sections were rinsed 3 × in 0.02% Tween in PBS, followed by 2 rinses in PBS and coverslipped with Vectashield Vibrance (Vector Labs, H-1700). Images were taken at 100 × magnification on an Olympus microscope using VS120 virtual scanner software (VS-ASW FL v. 2.7, Olympus Corporation, Tokyo, Japan).

### Statistics

This study represented a subcohort of a much larger swine cohort that was part of a multi-year swine study focusing on LDR effects across different tissues. Preliminary power analyses from previous investigations consistently showed that the sample size used in this study, or even smaller as in our previous investigation, was sufficient to obtain significant results considering the variability of the measurements performed in these studies. As for data distribution, Normality/Lognormality tests showed that the data distribution were mainly of gaussian type (D’Agostino and Pearson, Anderson–Darling, Shapiro–Wilk, and Kolmogorov-Smirnow tests all showed a higher probability for a normal distribution). We did not perform multiple comparisons since only 2 conditions (RAD vs. SH) for each single brain region were separately considered.

For each antibody and each examined region, two-tailed unpaired *t*-tests were performed for WB analyses. Data values are reported as mean + /−SEM. Differences between RAD versus SH group with *p*-value ≤ 0.05 were considered significant in all cases. Statistical tests were performed using GraphPad Prism version 9.0.2 for Windows (GraphPad Software, La Jolla, CA).

### Ethics approval

All animal work was approved by the Institutional Animal Care and Use Committee at the Uniformed Services University (USU, Bethesda, MD, USA) in compliance with the PHS Policy on Humane Care and Use of Laboratory Animals, the NIH Guide for the Care and Use of Laboratory Animals, and all applicable Federal regulations governing the protection of animals in research as well as in compliance with the ARRIVE guidelines (https://arriveguidelines.org/arrive-guidelines). The opinions expressed herein are those of the authors and not necessarily representative of those of the Uniformed Services University of the Health Sciences (USUHS), the Department of Defense (DOD) or the United States Army, Navy, or Air Force or any other US government agency and Henry M. Jackson Foundation for the Advancement of Military Medicine, Inc. (HJF).

### Supplementary Information

Supplementary Figures.

## Data Availability

The datasets used and/or analyzed during the current study and supporting the conclusions of this article are included in this article and in all supplementary materials provided. These datasets are also available from the corresponding author on reasonable request.
