# Supplementary material for: Low-dose brain radiation: lowering hyperphosphorylated-tau without increasing DNA damage or oncogenic activation
Source: Sci Rep. 2023 Nov 30;13:21142. doi: 10.1038/s41598-023-48146-w (PMC10689500; doi:10.1038/s41598-023-48146-w)
Supplement: Supplementary file 1 — Supplementary Figures. [file 41598_2023_48146_MOESM1_ESM.pdf]

Suppl. Fig. 1

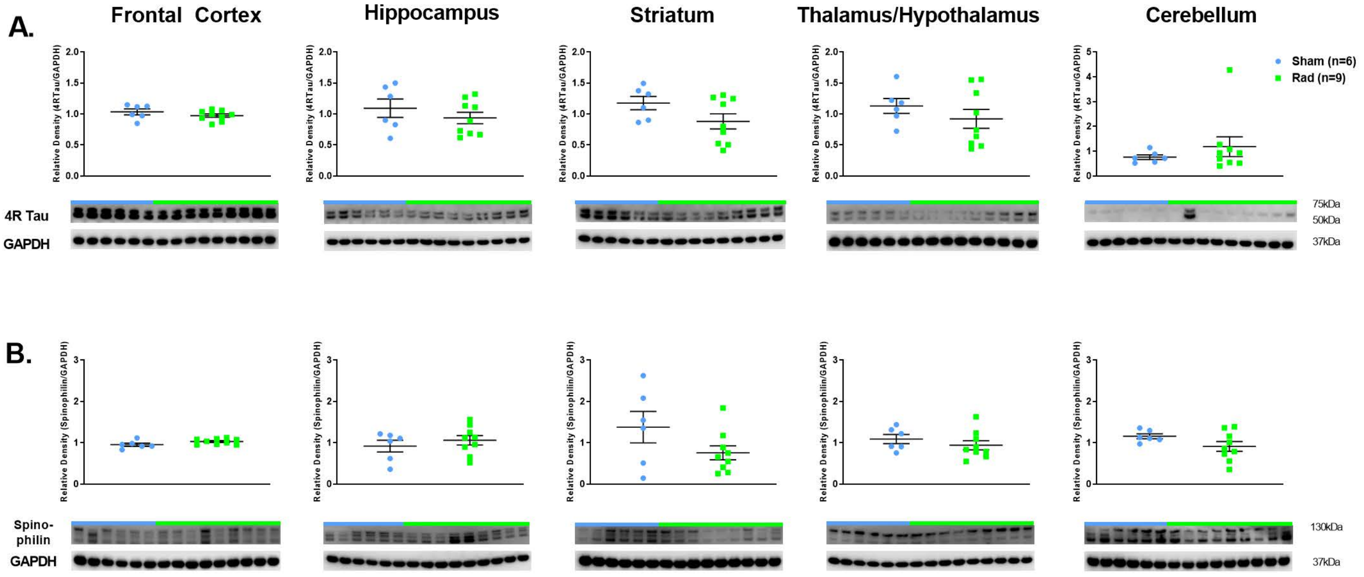

Supplemental Figure 1. Western blot densitometric analysis and representative blots for 4RTau (A.) and Spinophilin (B.) in Frontal Cortex, Hippocampus, Striatum, Thalamus/Hypothalamus and Cerebellum from the brains of Sham (blue) and Irradiated (green) swine 33-35 days post total body radiation. There were no significant differences to report. Each blot was run in duplicate and the graphs represent the average of 2 runs. Original full-length blots are presented in Supplementary Figures 18-19.

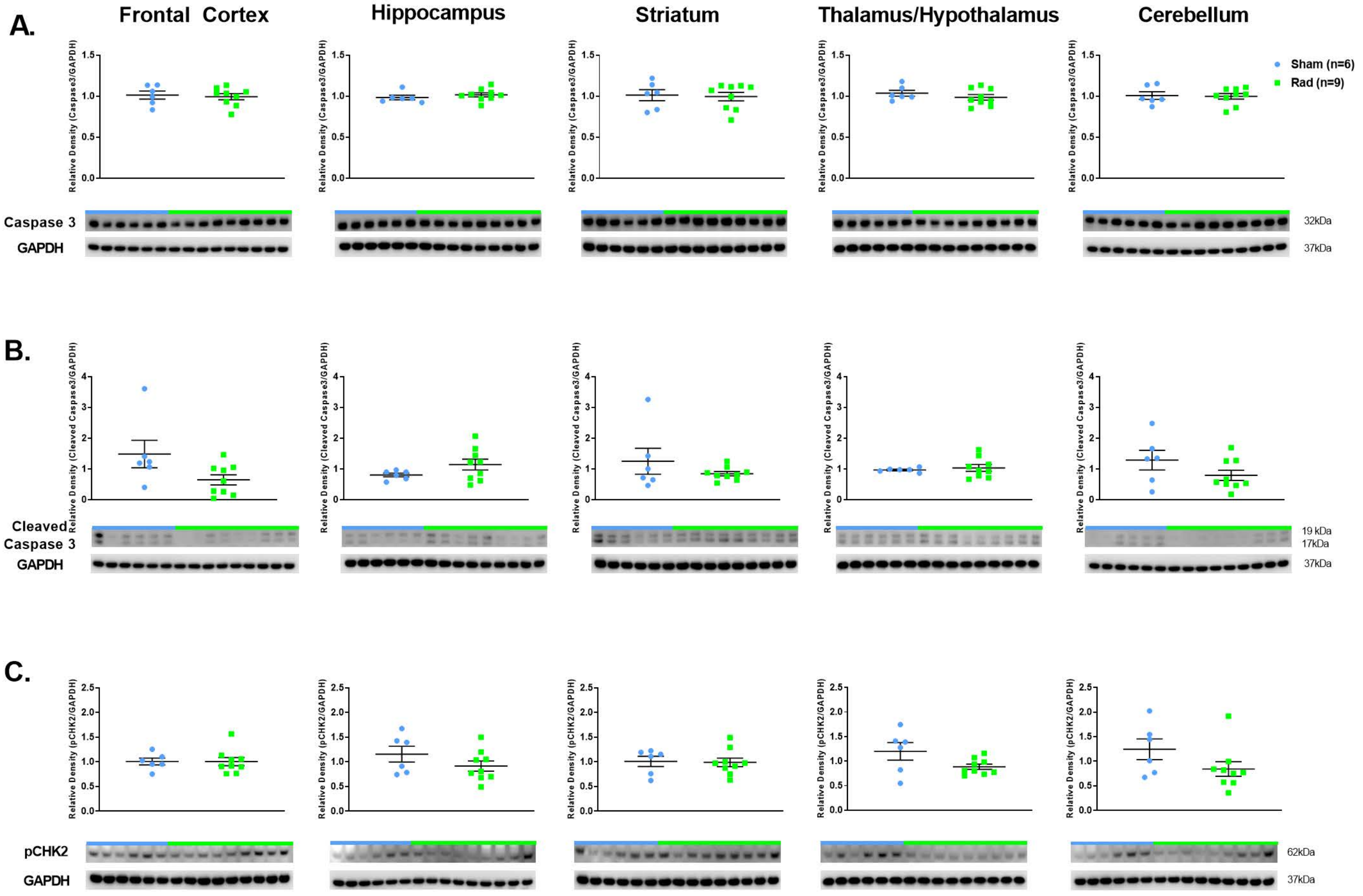

Supplemental Figure 2. Western blot densitometric analysis and representative blots for Caspase-3 (A.), Cleaved Caspase-3 (B.) and pCHK (C.) in Frontal Cortex, Hippocampus, Striatum, Thalamus/Hypothalamus and Cerebellum from the brains of Sham (blue) and Irradiated (green) swine 33-35 days post total body radiation. There were no significant differences to report. Each blot was run in duplicate and the graphs represent the average of 2 runs. Original full-length blots are presented in Supplementary Figures 20-21.

Suppl. Fig 3

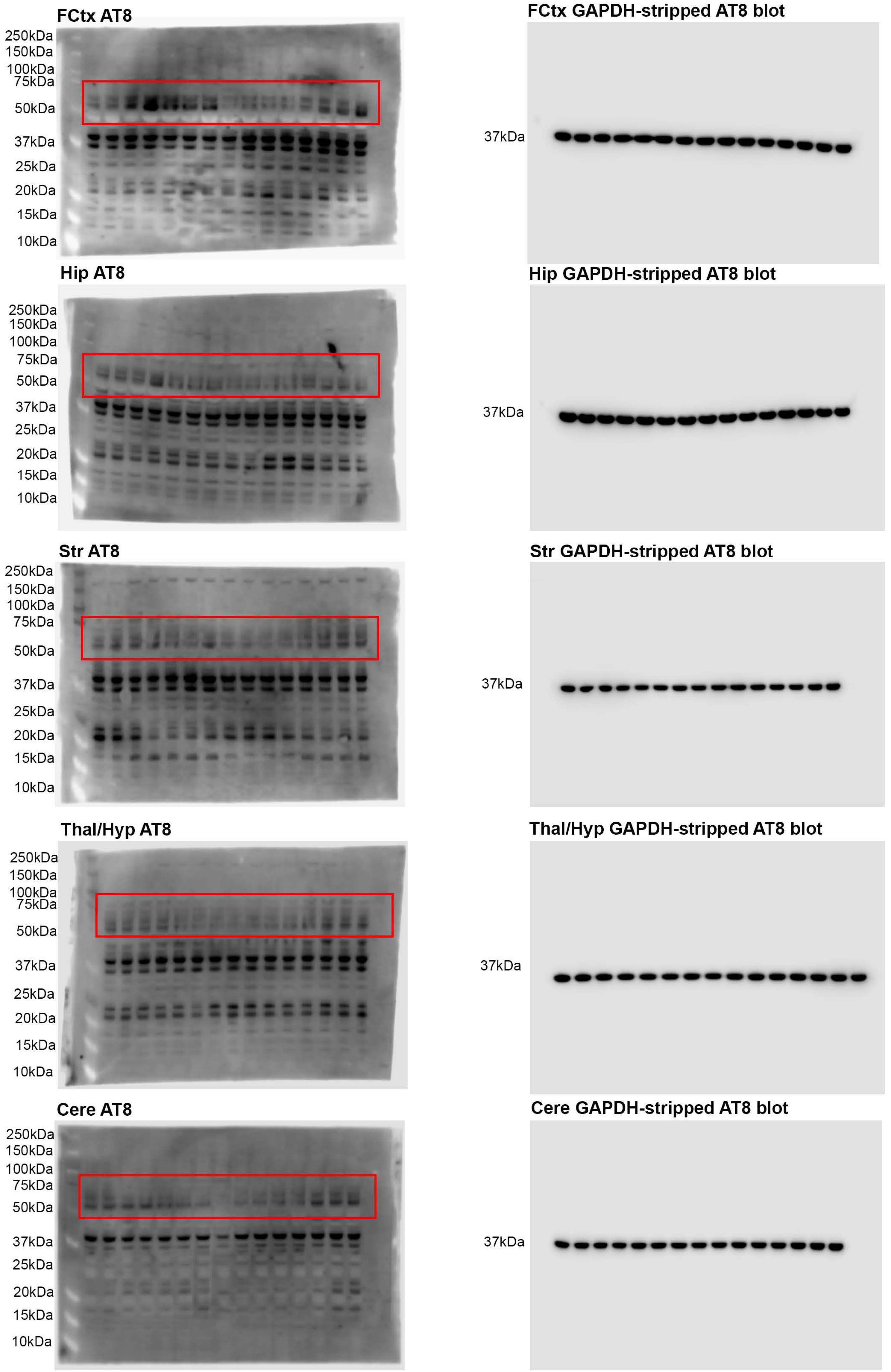

Supplemental Figure 3. Full blot images for AT8 and GAPDH for each examined region. Full blots correspond to the cropped images in Figure 1A.

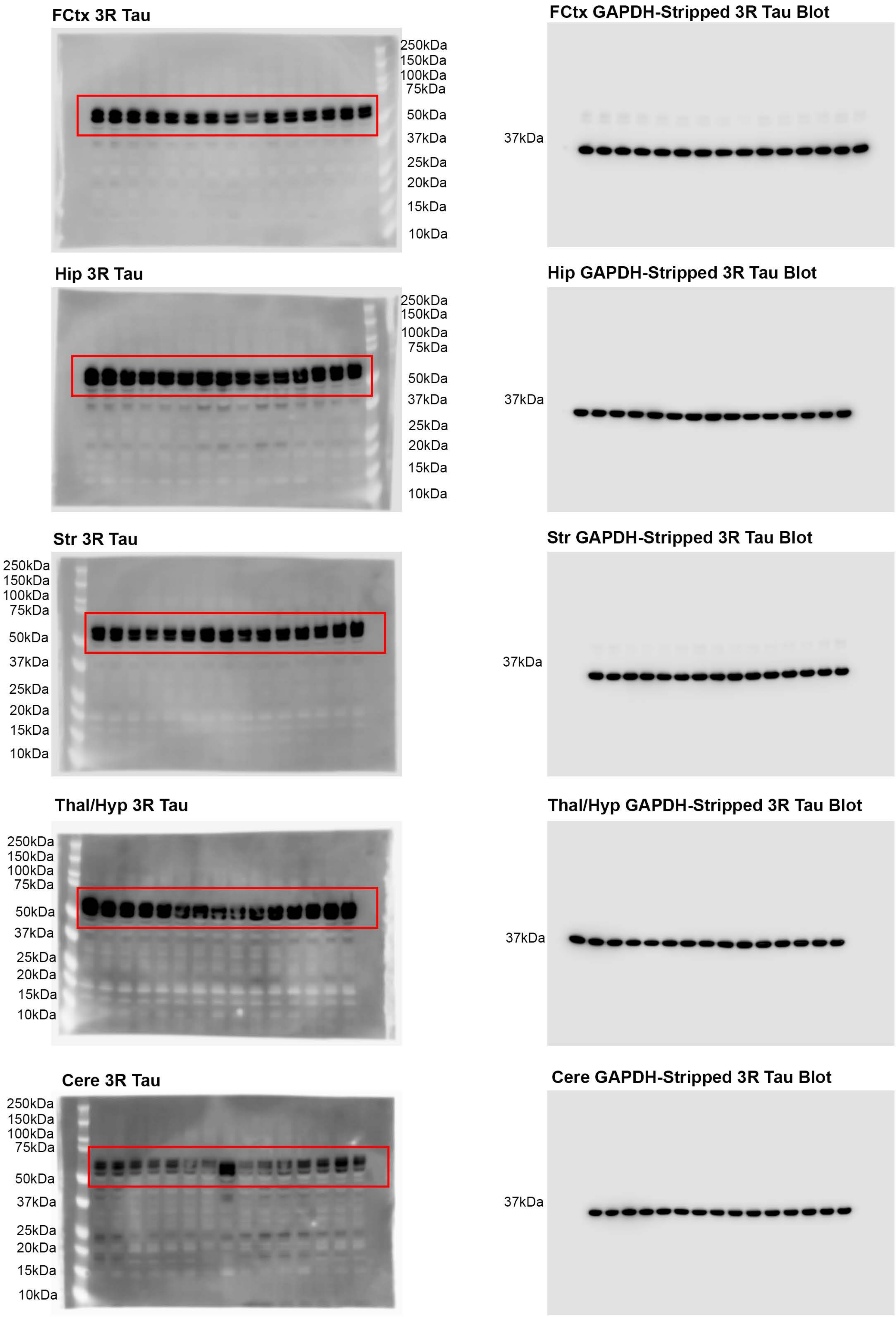

Supplemental Figure 4. Full blot images for 3RTau and GAPDH for each examined region. Full blots correspond to the cropped images in Figure 1B.

Suppl. Fig. 5

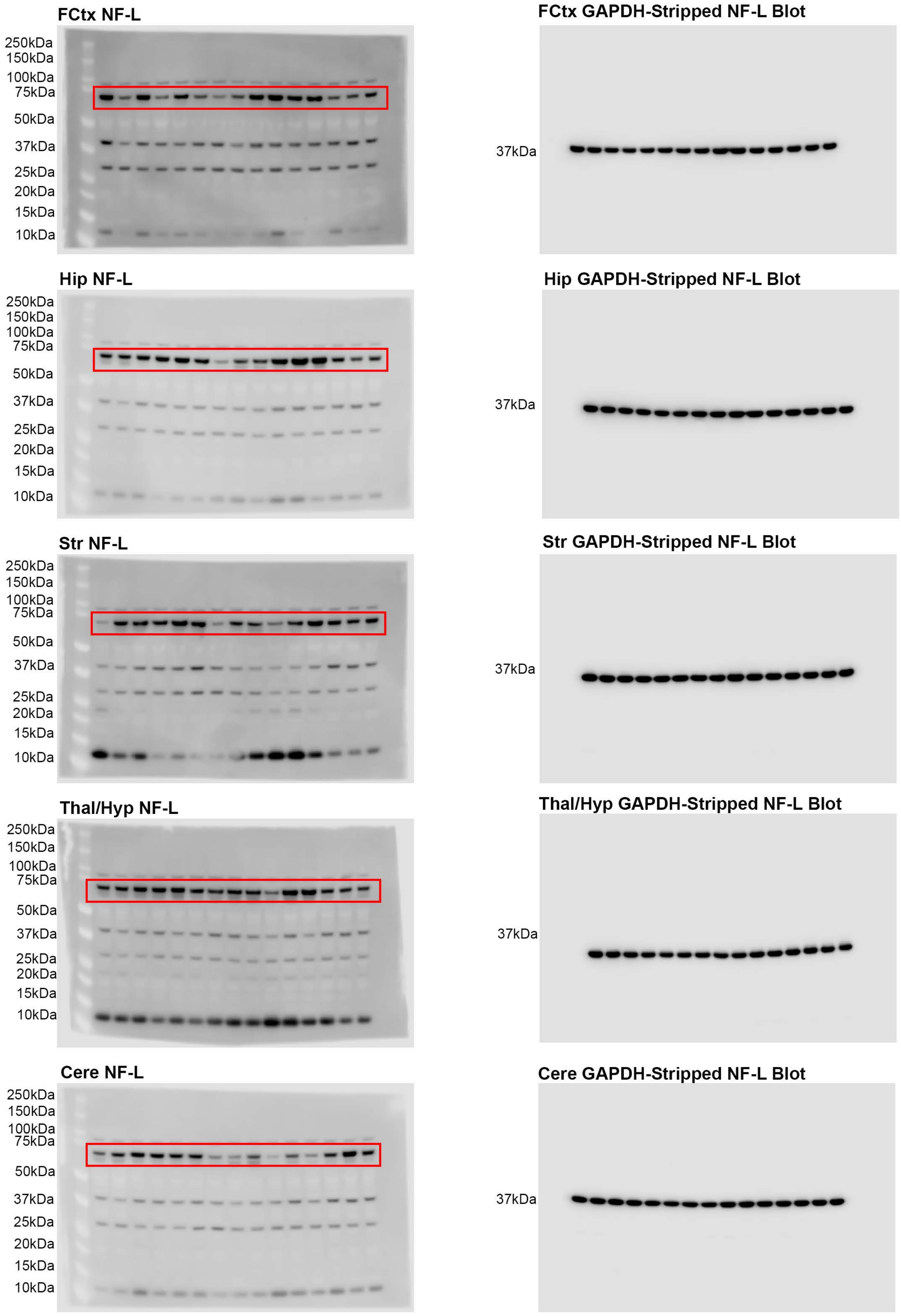

Supplemental Figure 5. Full blot images for NF-L and GAPDH for each examined region. Full blots correspond to the cropped images in Figure 1C.

Suppl. Fig. 6

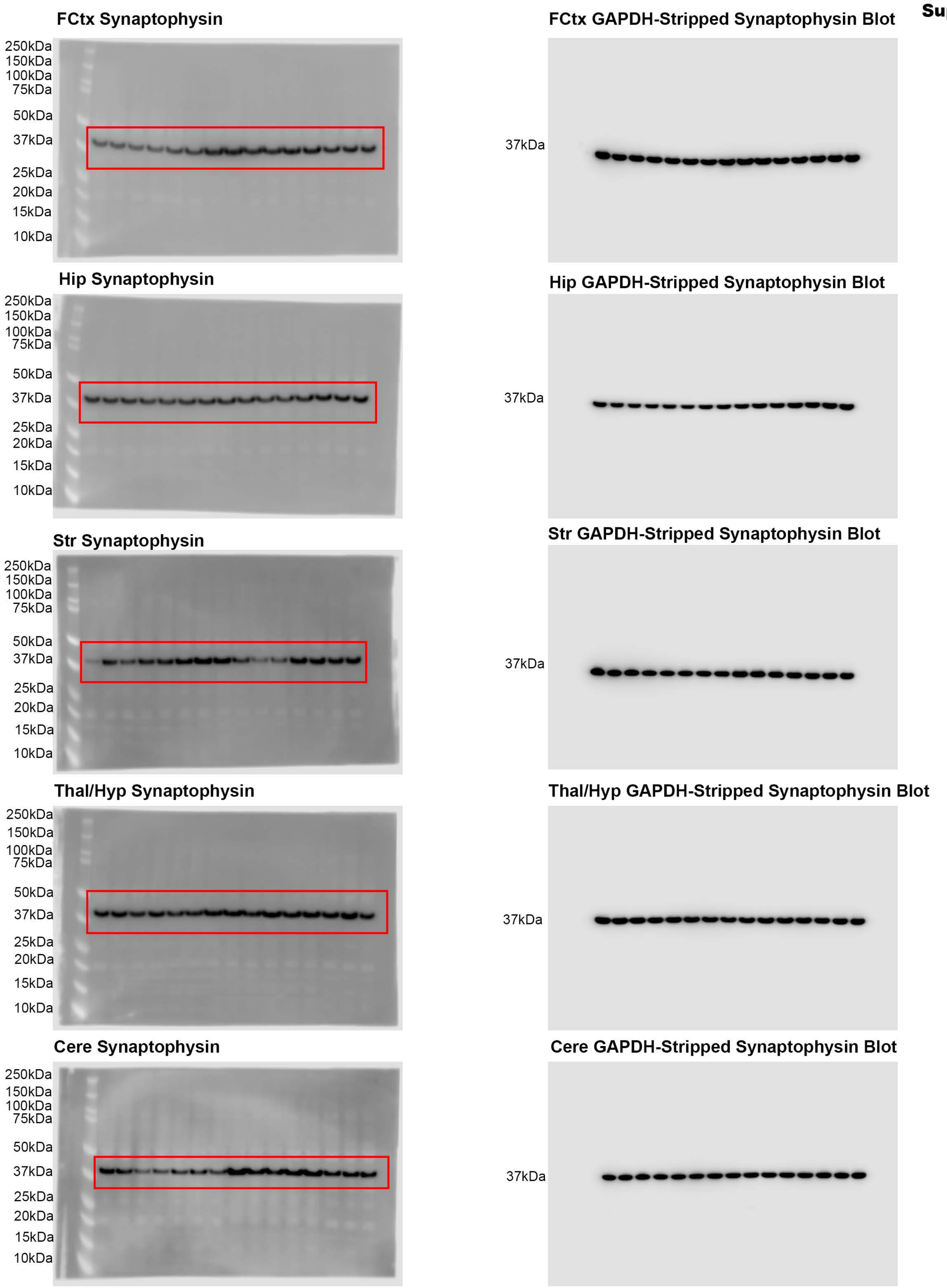

Supplemental Figure 6. Full blot images for Synaptophysin and GAPDH for each examined region. Full blots correspond to the cropped images in Figure 2A.

Suppl. Fig. 7

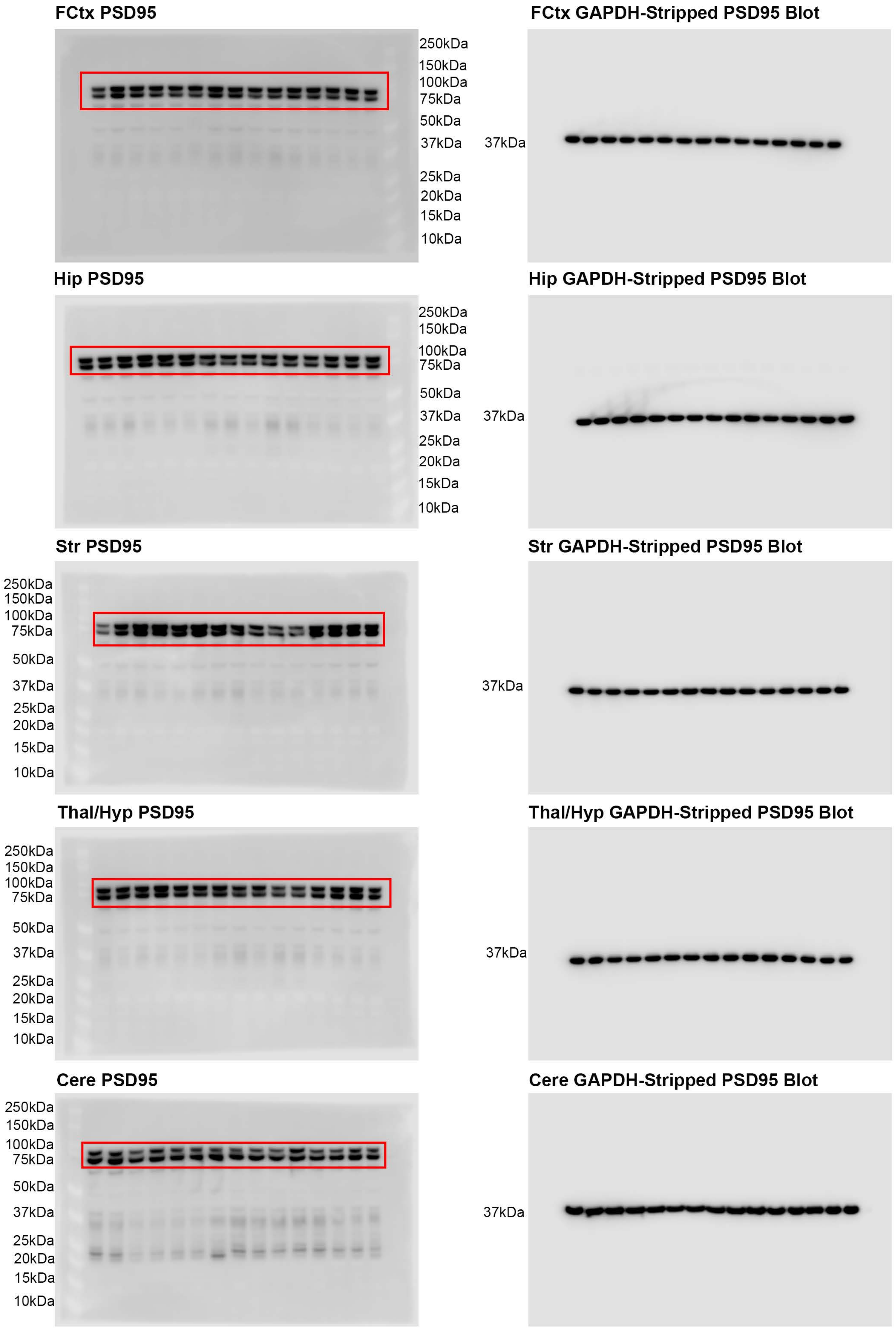

Supplemental Figure 7. Full blot images for PSD-95 and GAPDH for each examined region. Full blots correspond to the cropped images in Figure 2B.

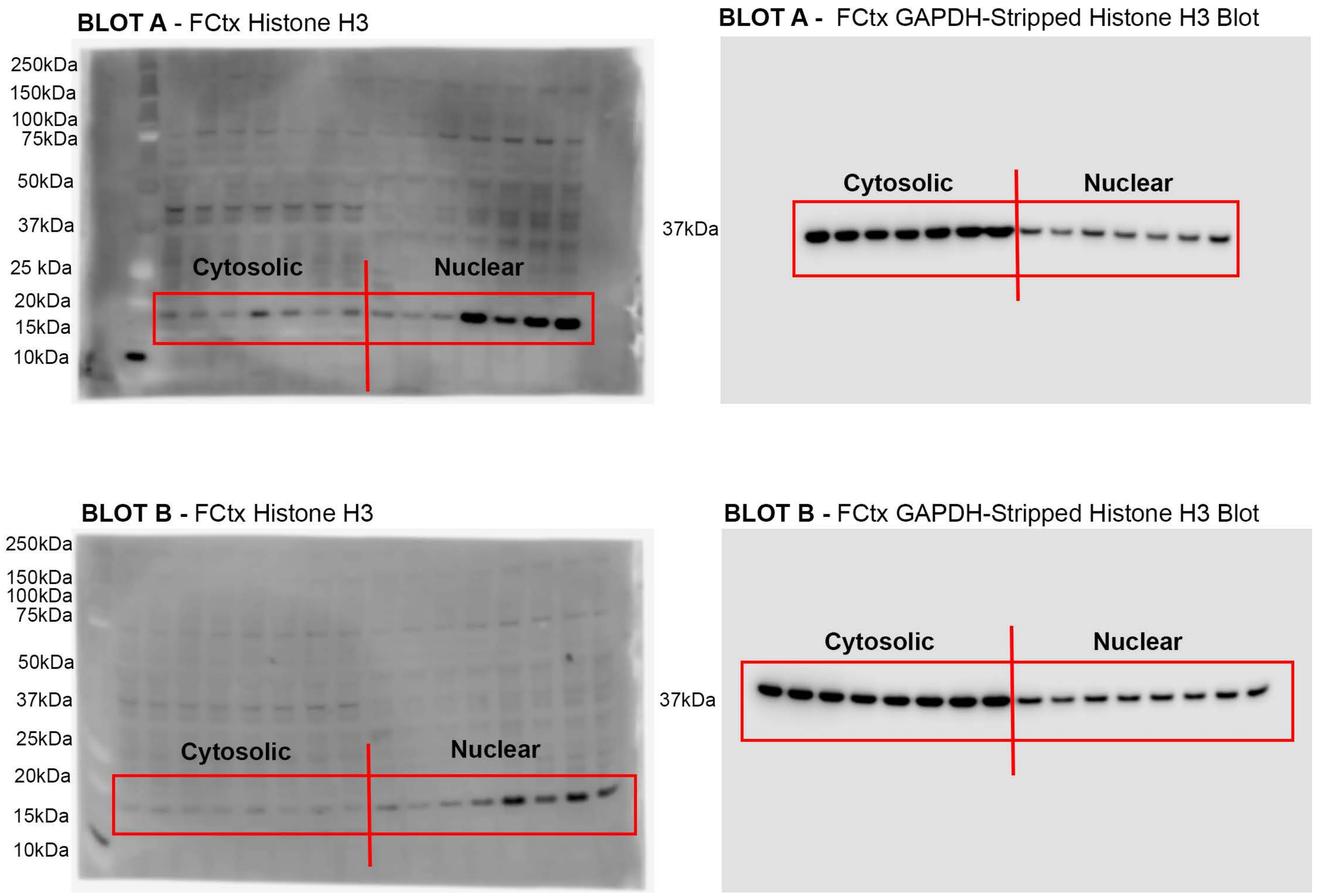

Supplemental Figure 8. Full blot images for Histone-H3 and GAPDH for **FCtx** Cytosolic and Nuclear extracts. Full blots correspond to the **FCtx** cropped images in Figure 3, CYT and NUC.

BLOT A - Hip Histone H3

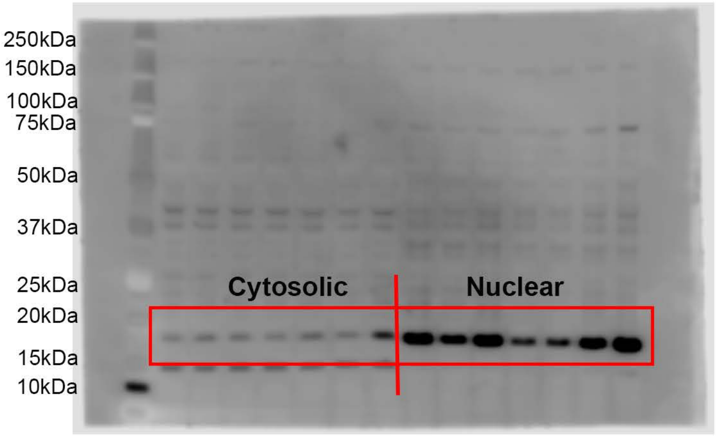

BLOT A - Hip GAPDH-Stripped Histone H3 Blot

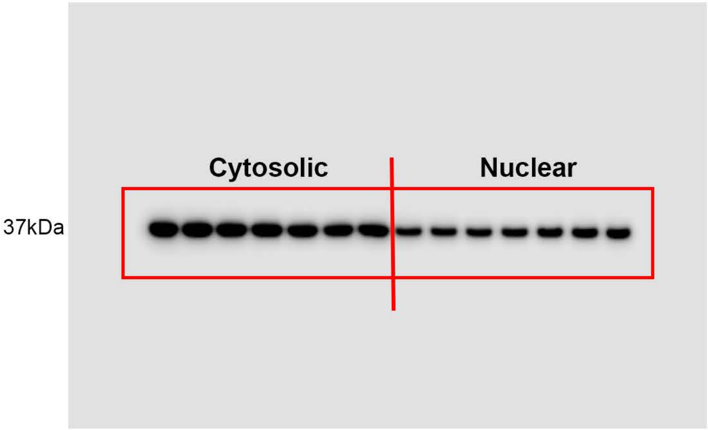

BLOT B - Hip Histone H3

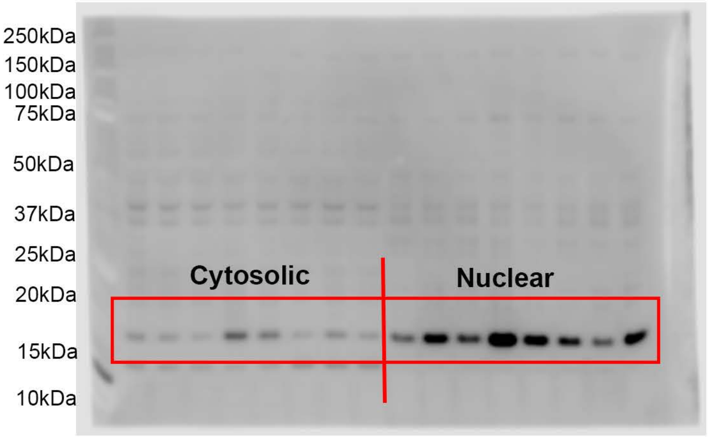

BLOT B - Hip GAPDH-Stripped Histone H3 Blot

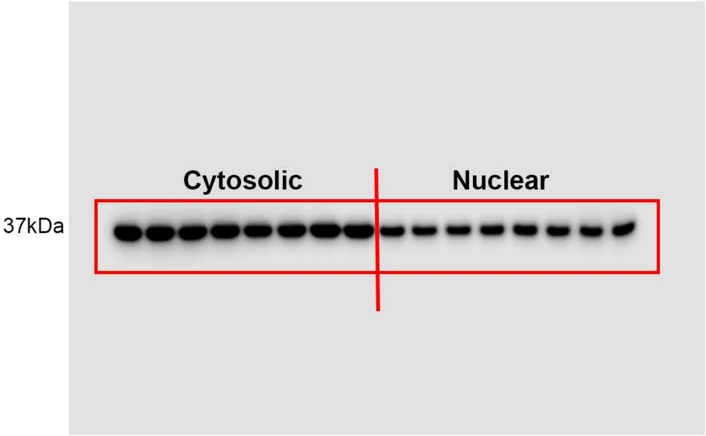

Supplemental Figure 9. Full blot images for Histone-H3 and GAPDH for **Hip** Cytosolic and Nuclear extracts. Full blots correspond to the **Hip** cropped images in Figure 3, CYT and NUC.

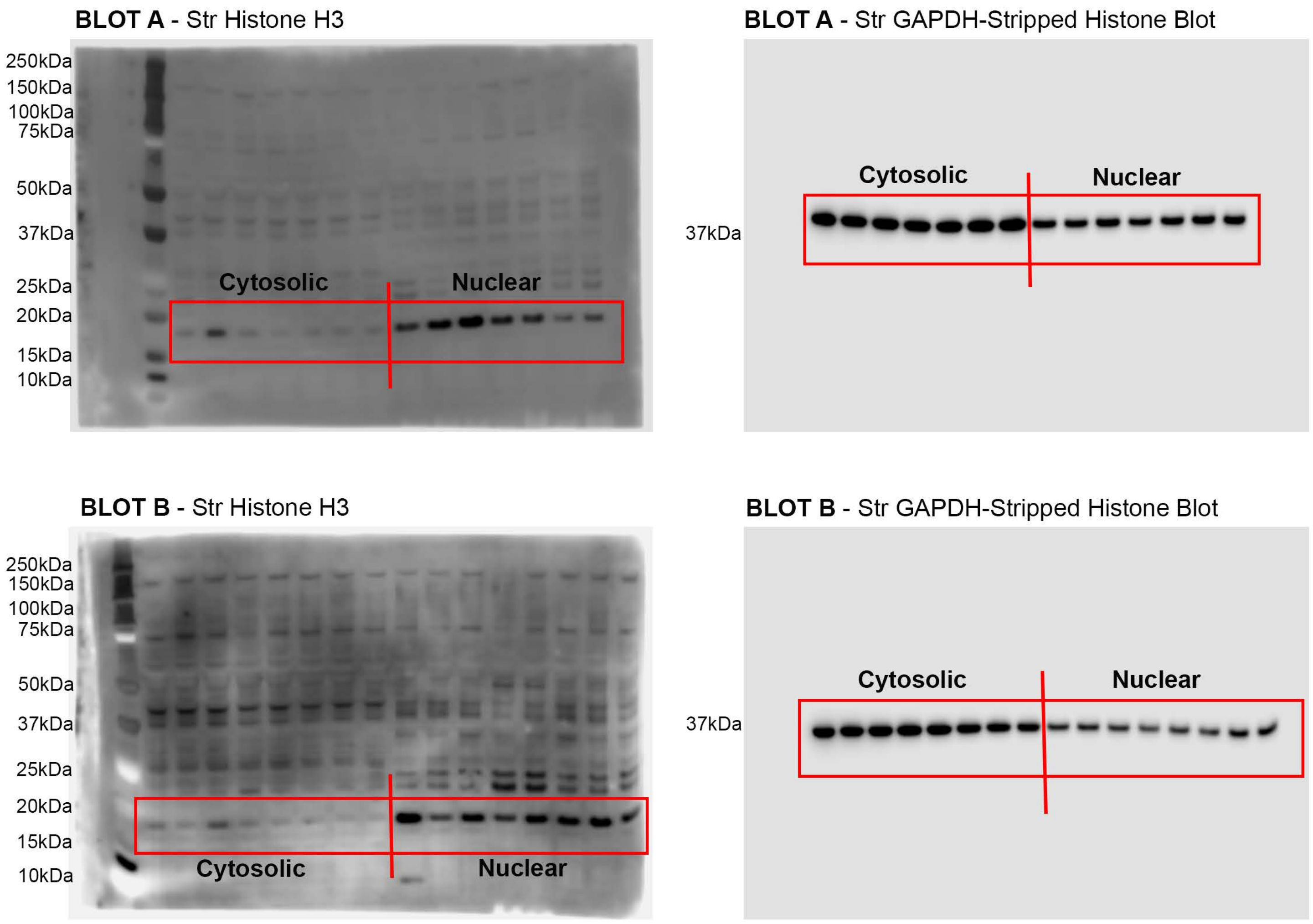

Supplemental Figure 10. Full blot images for Histone-H3 and GAPDH for **Str** Cytosolic and Nuclear extracts. Full blots correspond to the **Str** cropped images in Figure 3, CYT and NUC.

BLOT A - Thal/Hyp Histone H3

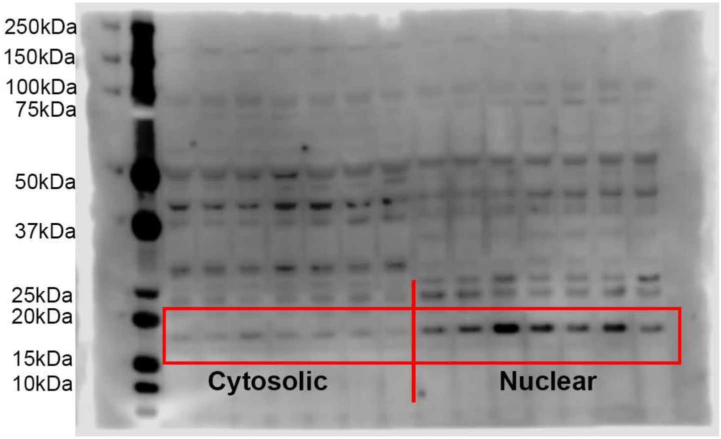

BLOT A - Thal/Hyp GAPDH-Stripped Histone H3 Blot

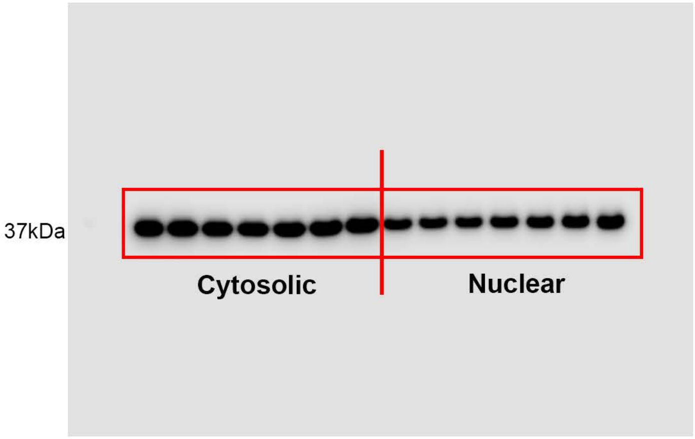

BLOT B - Thal/Hyp Histone H3

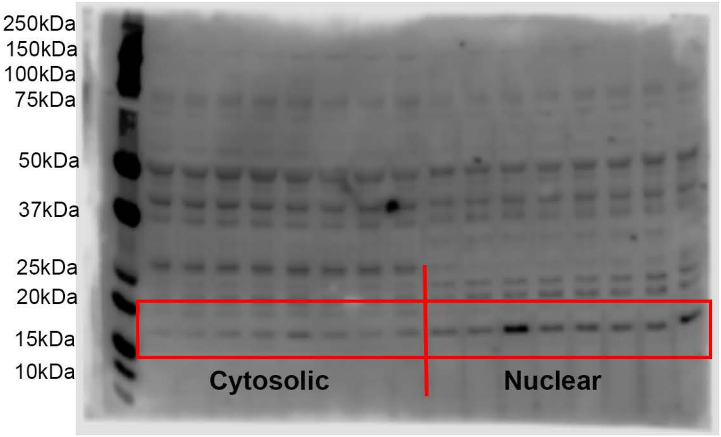

BLOT B - Thal/Hyp GAPDH-Stripped Histone H3 Blot

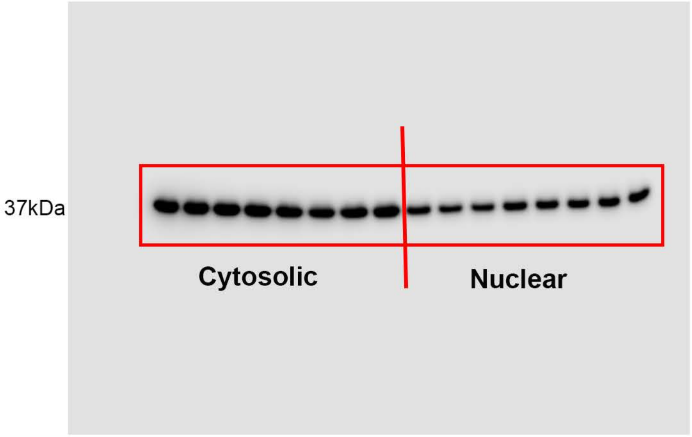

Supplemental Figure 11. Full blot images for Histone-H3 and GAPDH for **Thal/Hyp** Cytosolic and Nuclear extracts. Full blots correspond to the **Thal/Hyp** cropped images in Figure 3, CYT and NUC.

BLOT A - Cere Histone H3

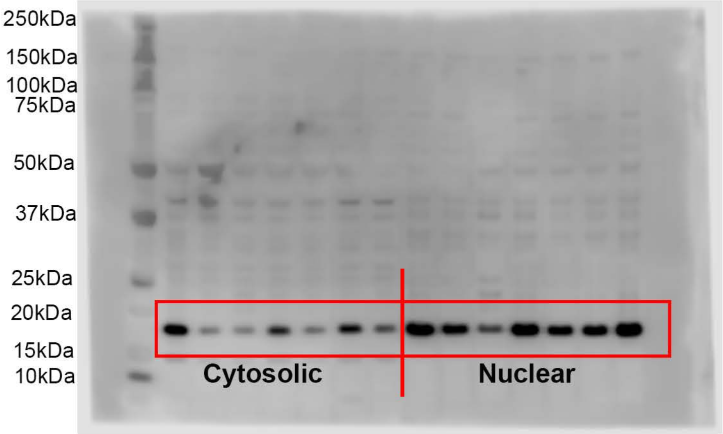

BLOT A - Cere GAPDH-Stripped Histone H3 Blot

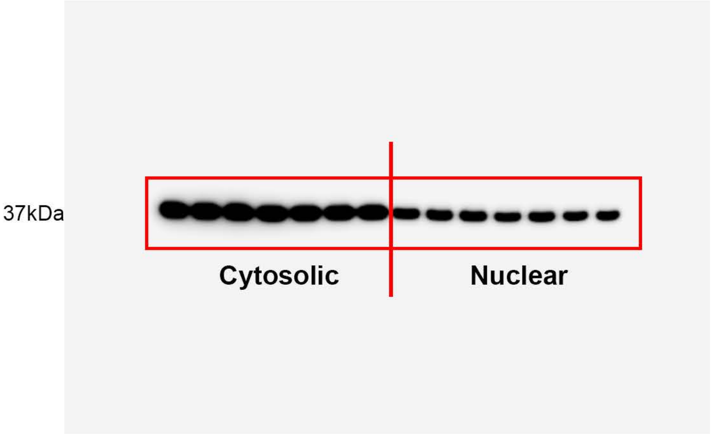

BLOT B - Cere Histone H3

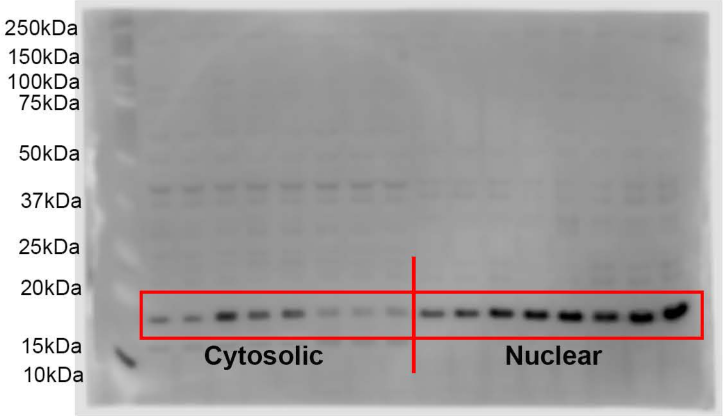

BLOT B - Cere GAPDH-Stripped Histone H3 Blot

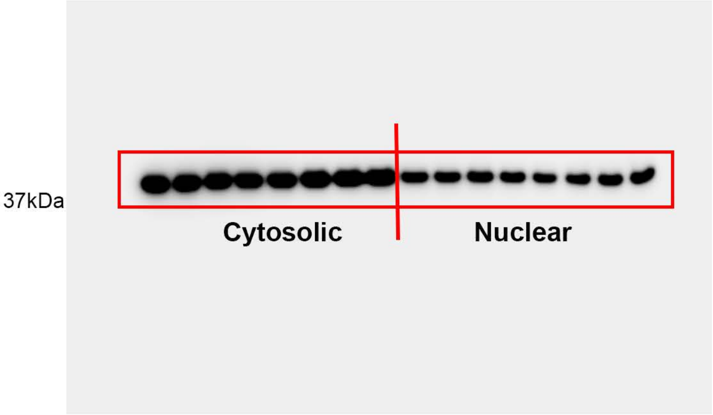

Supplemental Figure 12. Full blot images for Histone-H3 and GAPDH for **Cere** Cytosolic and Nuclear extracts. Full blots correspond to the **Cere** cropped images in Figure 3, CYT and NUC.

BLOT A - FCtx p53

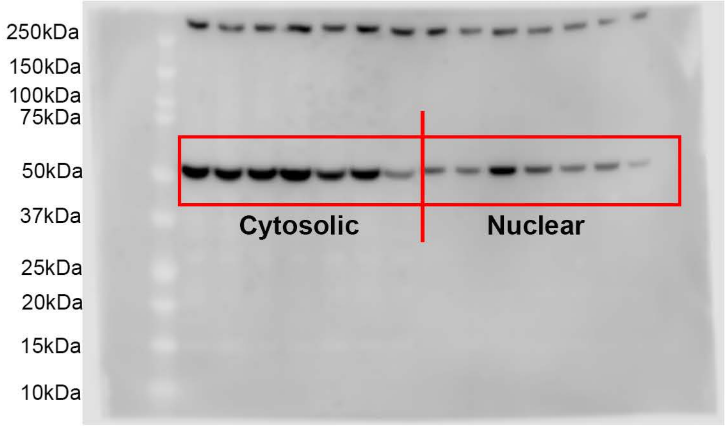

BLOT A - FCtx GAPDH-Stripped p53 Blot

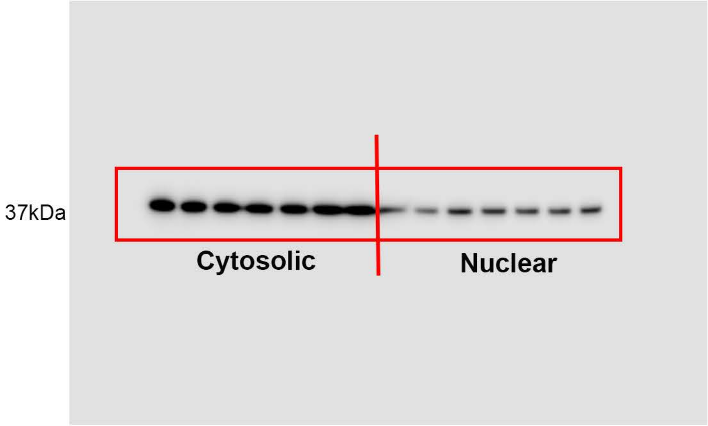

BLOT B - FCtx p53

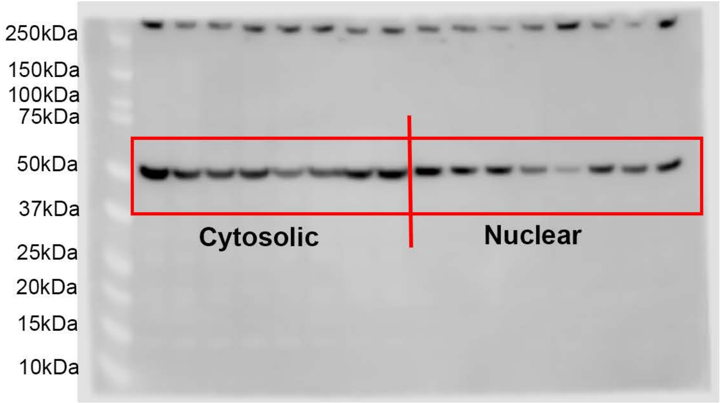

BLOT B - FCtx GAPDH-Stripped p53 Blot

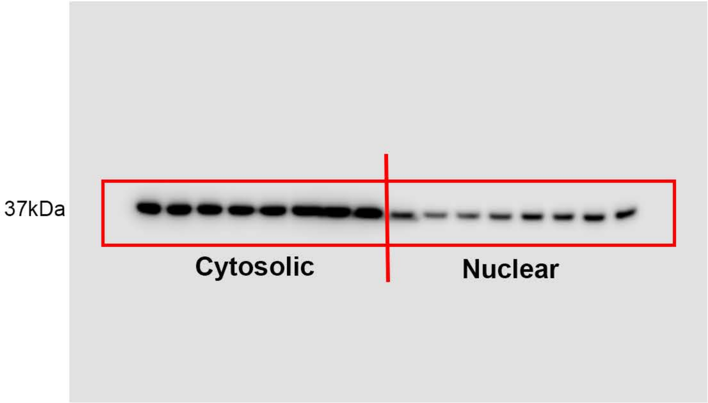

Supplemental Figure 13. Full blot images for p53 and GAPDH for **FCtx** Cytosolic and Nuclear extracts. Full blots correspond to the **FCtx** cropped images in Figure 4, CYT and NUC.

Suppl. Fig. 14

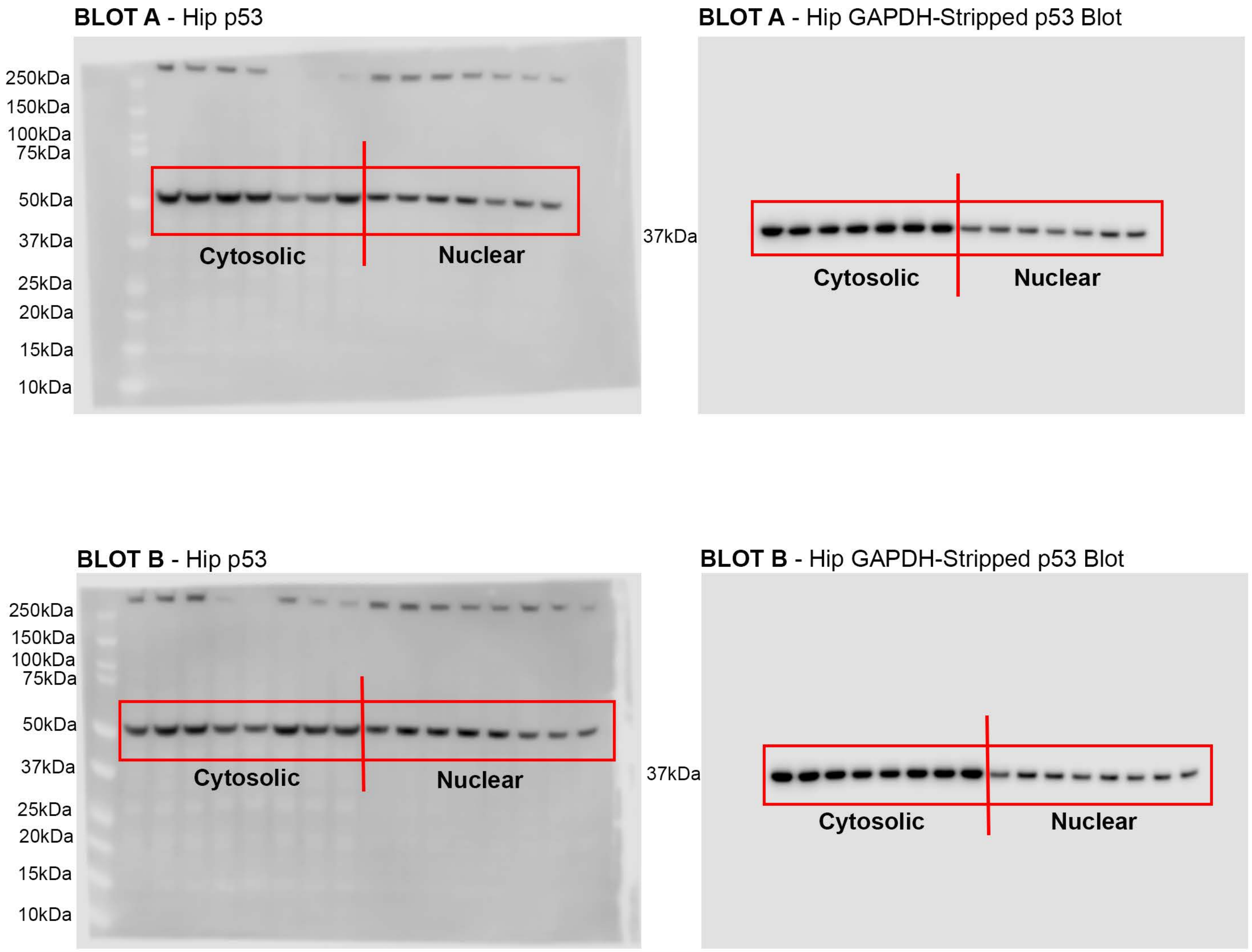

Supplemental Figure 14. Full blot images for p53 and GAPDH for **Hip** Cytosolic and Nuclear extracts. Full blots correspond to the **Hip** cropped images in Figure 4, CYT and NUC.

BLOT A - Str p53

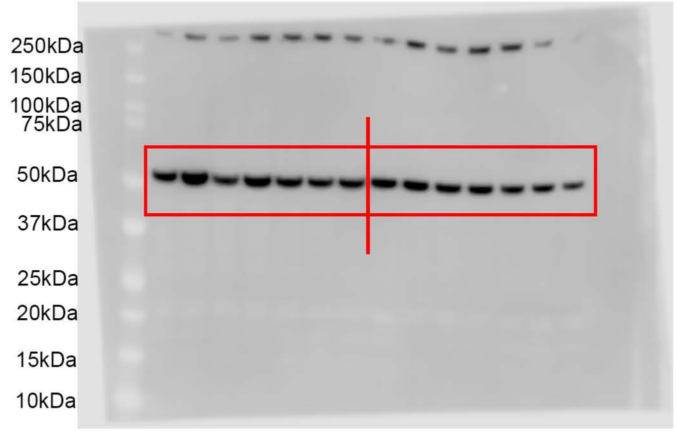

BLOT A - Str GAPDH-Stripped p53 Blot

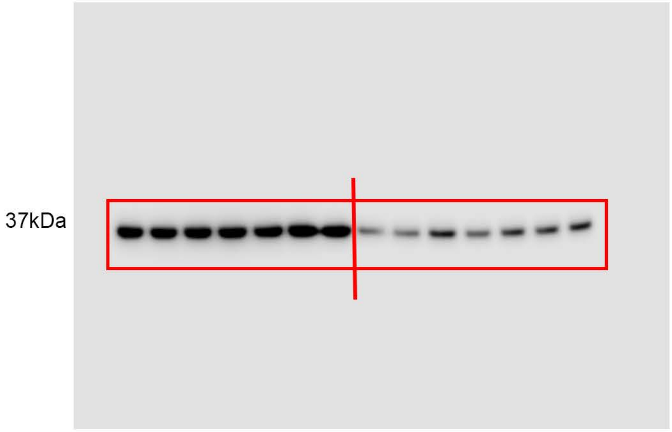

BLOT B - Str p53

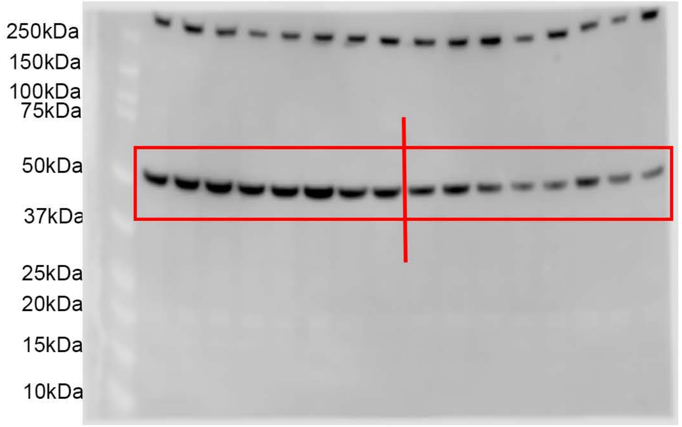

BLOT B - Str GAPDH-Stripped p53 Blot

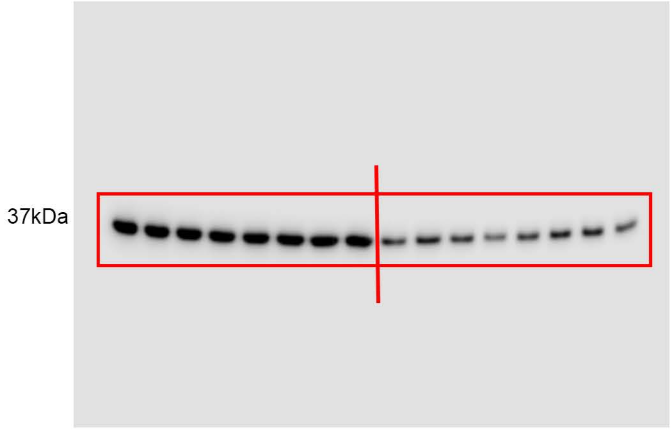

Supplemental Figure 15. Full blot images for p53 and GAPDH for **Str** Cytosolic and Nuclear extracts. Full blots correspond to the **Str** cropped images in Figure 4, CYT and NUC.

BLOT A - Thal-Hyp p53

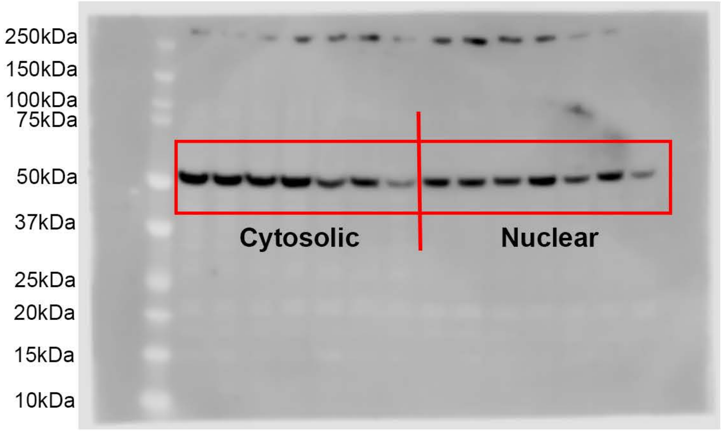

BLOT A - Thal-Hyp GAPDH-Stripped p53 Blot

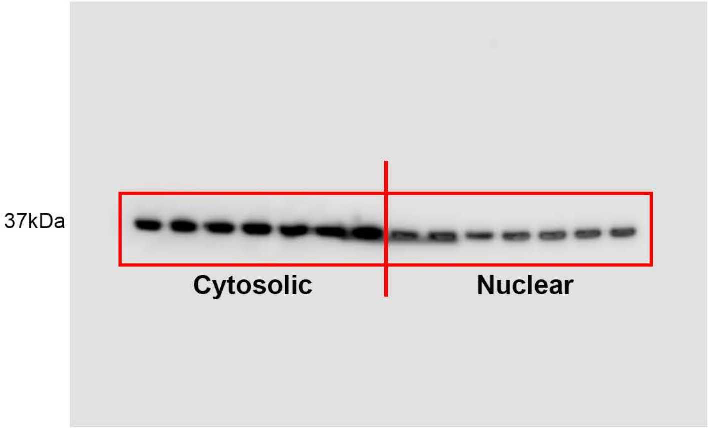

BLOT B - Thal-Hyp p53

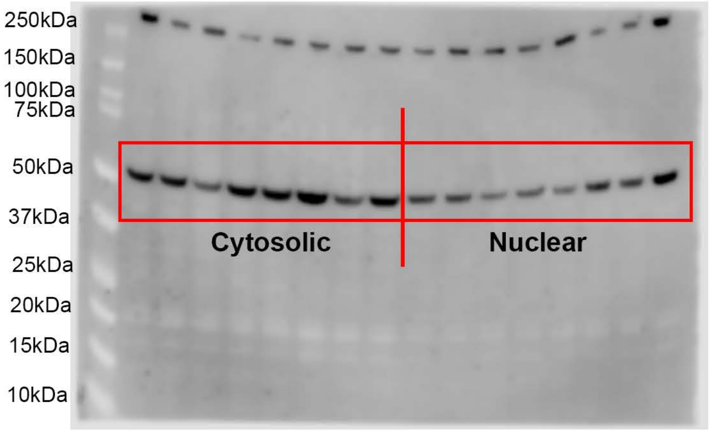

BLOT B - Thal-Hyp p53 GAPDH-Stripped p53 Blot

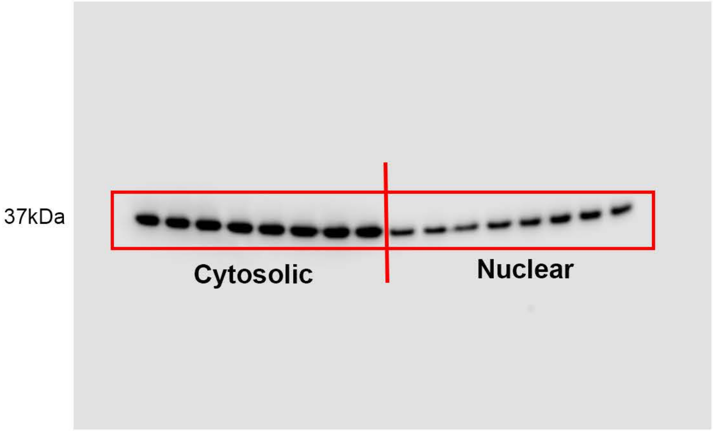

Supplemental Figure 16. Full blot images for p53 and GAPDH for **Thal/Hyp** Cytosolic and Nuclear extracts. Full blots correspond to the **Thal/Hyp** cropped images in Figure 4, CYT and NUC.

BLOT A - Cere p53

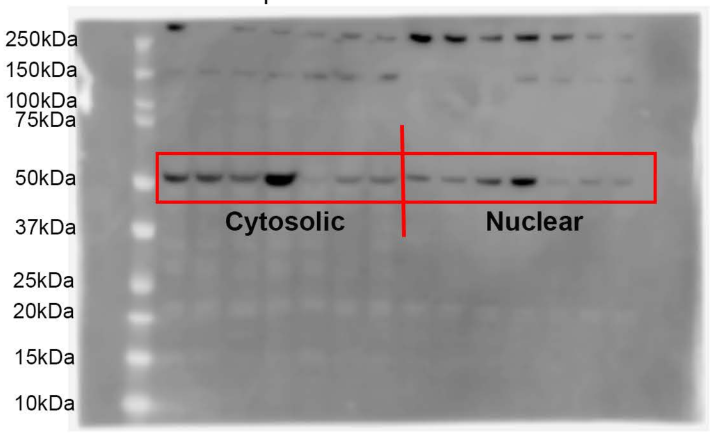

BLOT A - Cere GAPDH-Stripped p53 Blot

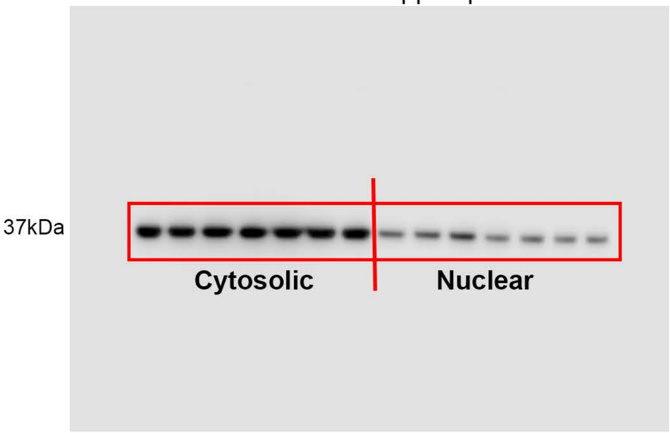

BLOT B - Cere p53

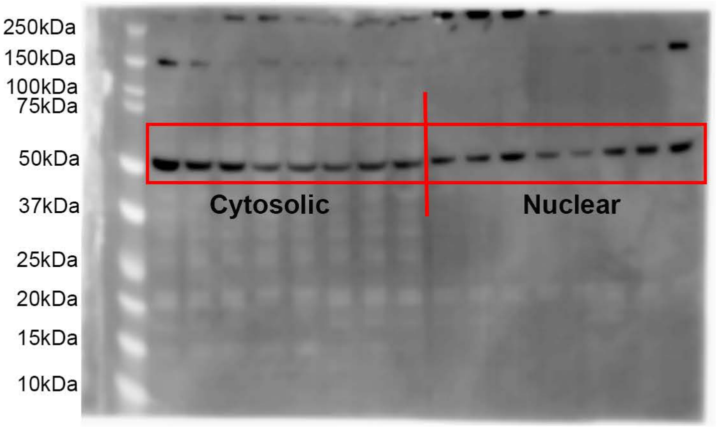

BLOT B - Cere GAPDH-Stripped p53 Blot

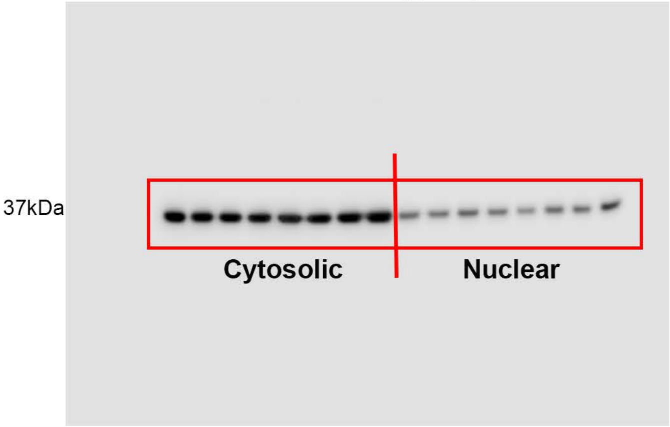

Supplemental Figure 17. Full blot images for p53 and GAPDH for **Cere** Cytosolic and Nuclear extracts. Full blots correspond to the **Cere** cropped images in Figure 4, CYT and NUC.

Suppl. Fig. 18

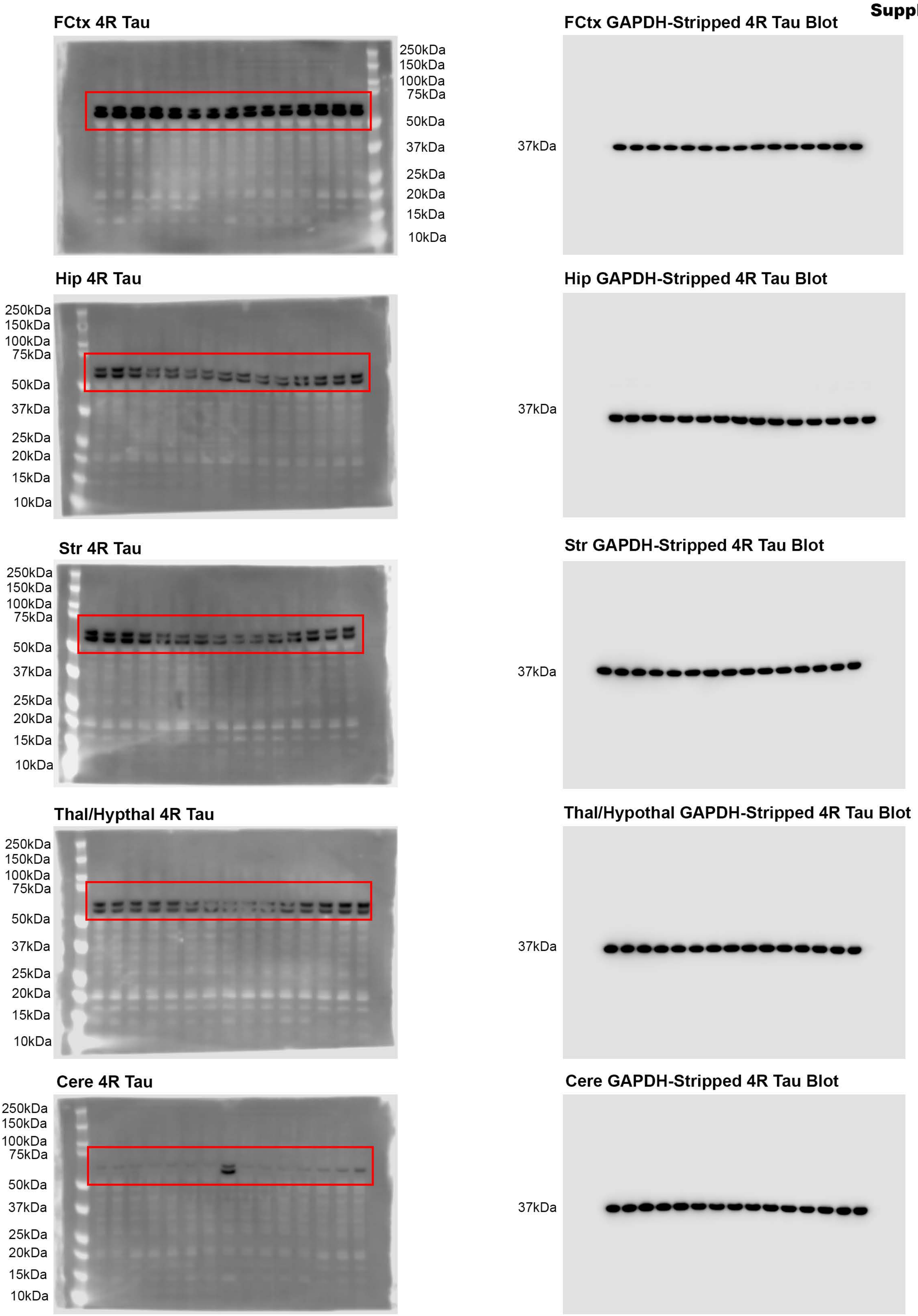

Supplemental Figure 18. Full blot images for 4RTau and GAPDH for each examined region. Full blots correspond to the cropped images in Supplemental Figure 1A.

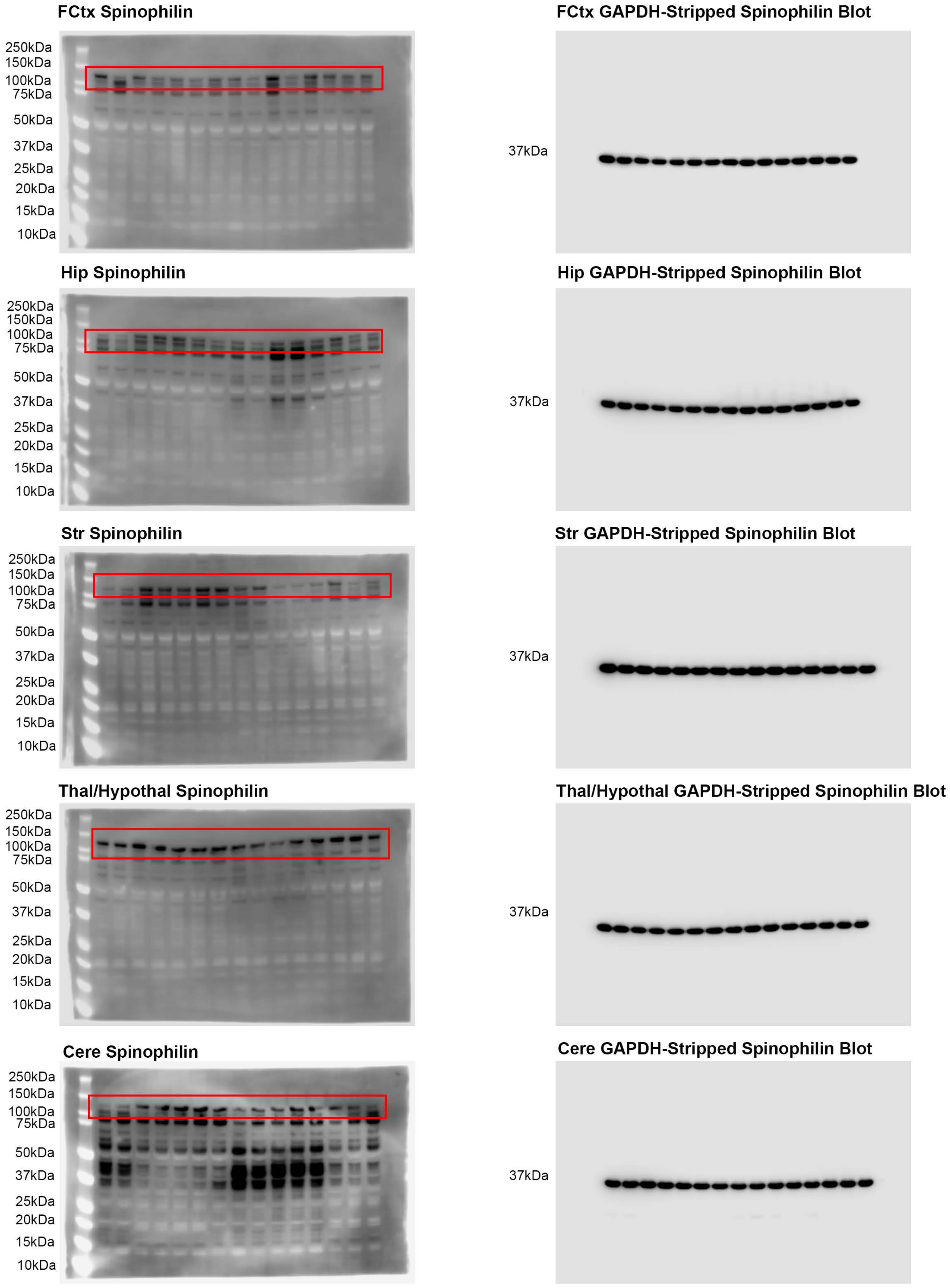

Supplemental Figure 19. Full blot images for Spinophilin and GAPDH for each examined region. Full blots correspond to the cropped images in Supplemental Figure 1B.

Suppl. Fig. 20

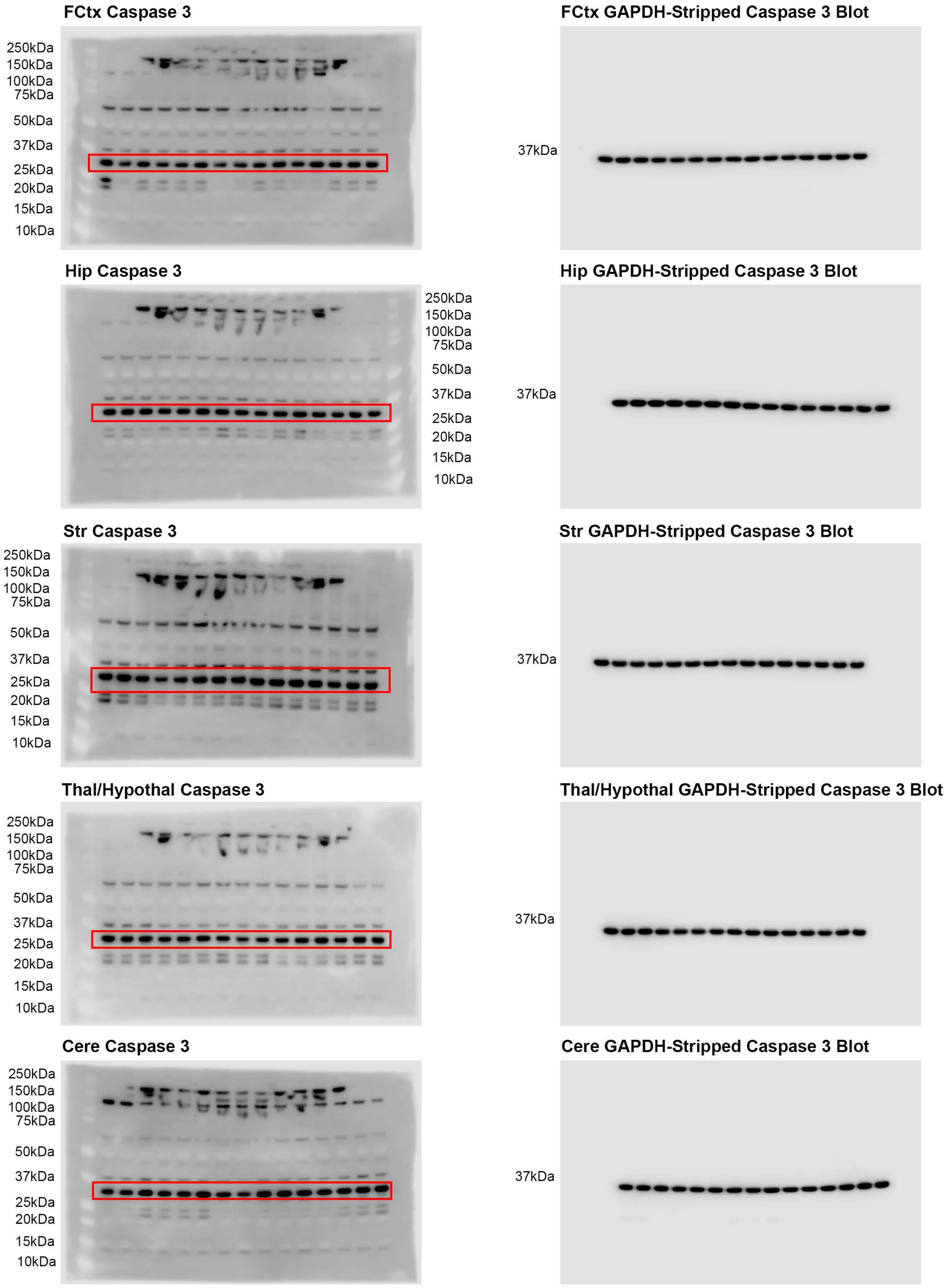

Supplemental Figure 20. Full blot images for Caspase3 and GAPDH for each examined region. Full blots correspond to the cropped images in Supplemental Figure 2A.

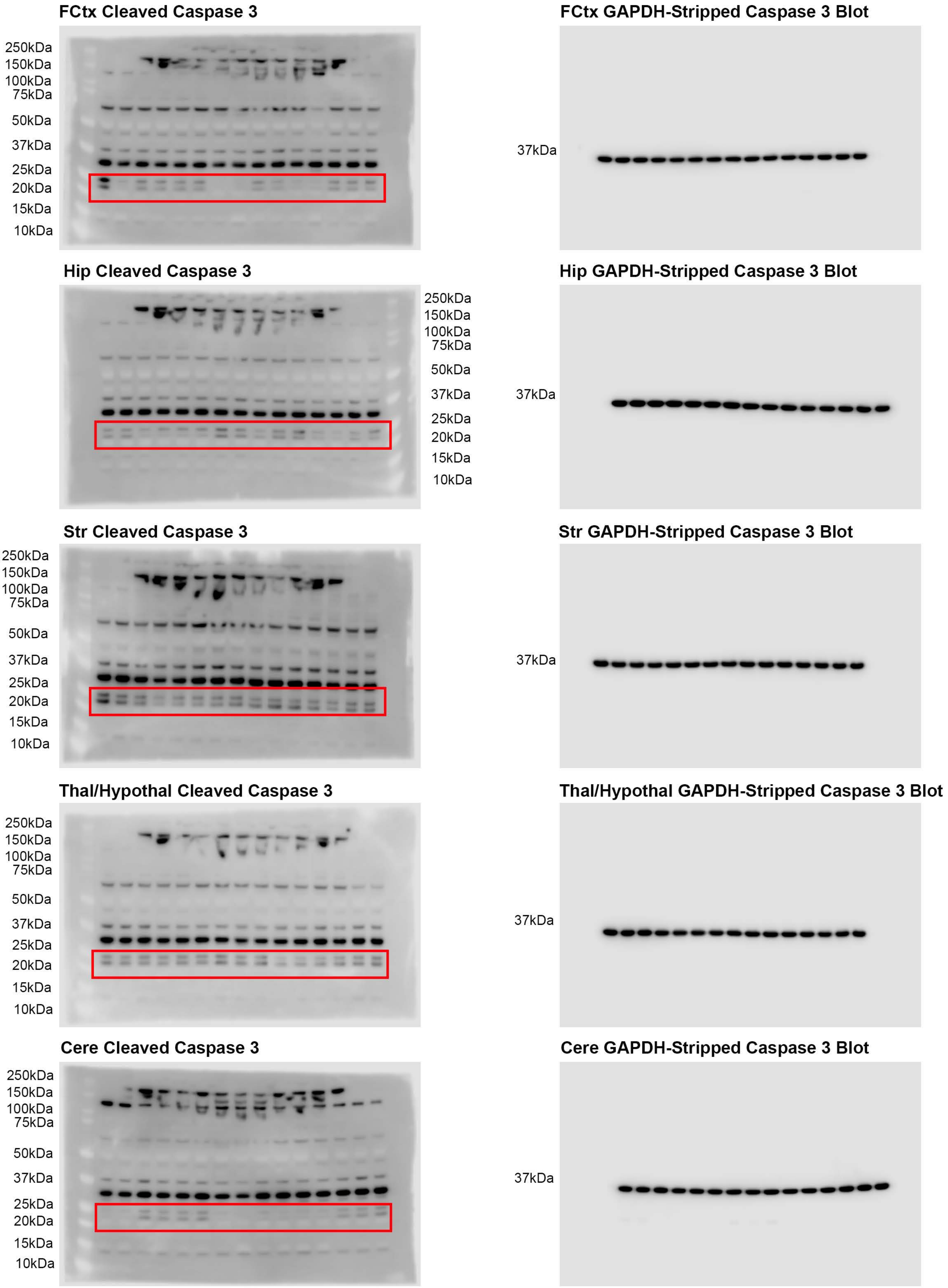

Supplemental Figure 21. Full blot images for Cleaved Caspase3 and GAPDH for each examined region. Full blots correspond to the cropped images in Supplemental Figure 2B.

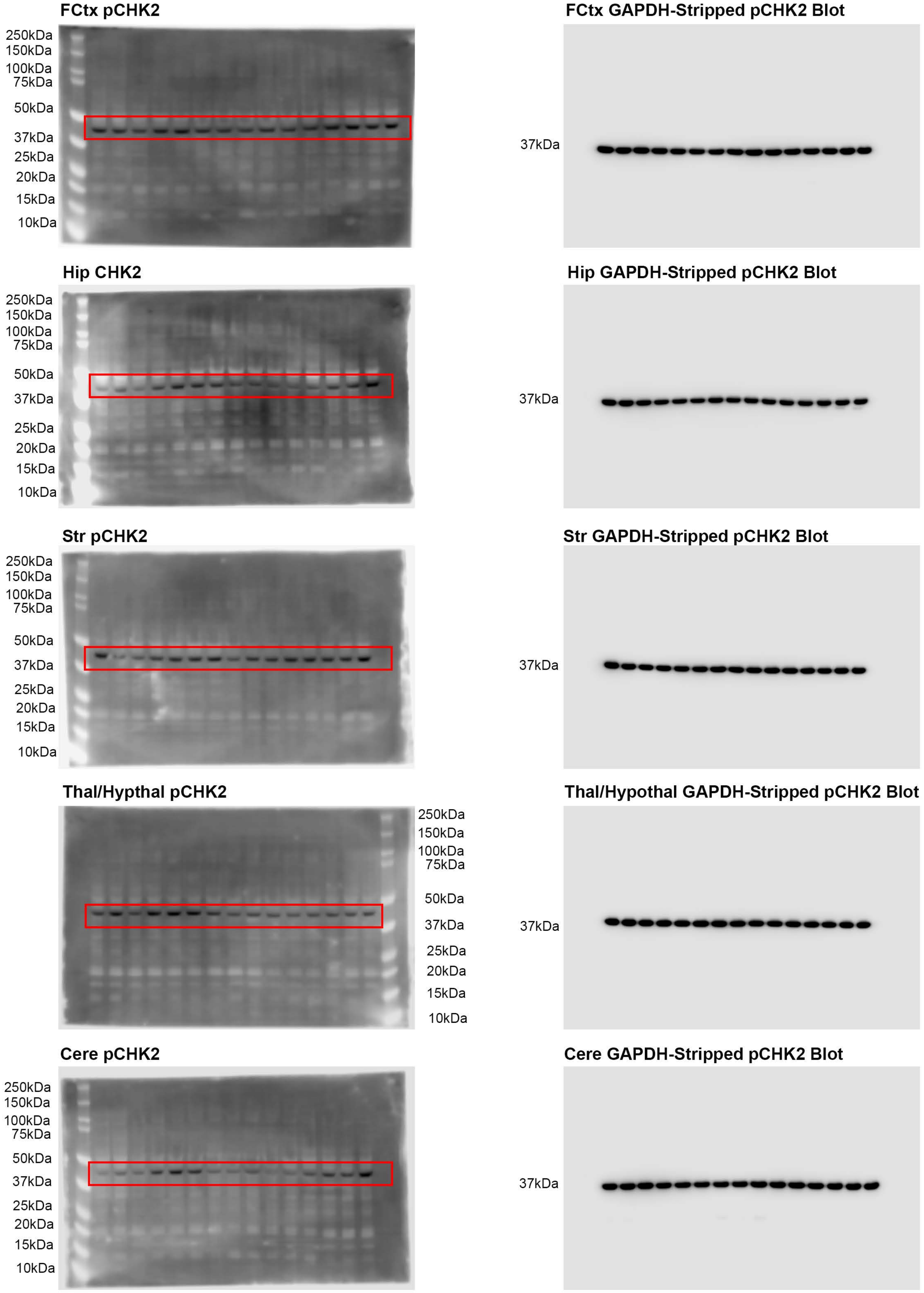

Supplemental Figure 22. Full blot images for pCHK2 and GAPDH for each examined region. Full blots correspond to the cropped images in Supplemental Figure 2C.
